# Decoding Preschool Social Dynamics: Automated Tracking of Spatial and Temporal Patterns to Investigate Social Interactions and Relationships in Peer Groups

**DOI:** 10.1111/desc.70241

**Published:** 2026-06-18

**Authors:** Gabriela Markova, Jozsef Arato, Ruzena Ceral, Maximilian Hofbauer, Cliodhna Quigley, Lisa Horn

**Affiliations:** ^1^ Faculty of Psychology University of Vienna Vienna Austria; ^2^ Institute for Early Life Care Paracelsus Medical Private University Salzburg Austria; ^3^ Department of Pediatrics University Hospital Salzburg Paracelsus Medical Private University Salzburg Austria; ^4^ Vienna Cognitive Science Hub University of Vienna Vienna Austria; ^5^ Department of Cognitive Science Budapest University of Technology and Economics Budapest Hungary; ^6^ Department of Behavioral and Cognitive Biology University of Vienna Vienna Austria; ^7^ Loopbio GmbH Vienna Austria

**Keywords:** automated behavior tracking, friendship, naturalistic observation, preschool, social interaction, spatio‐temporal proximity

## Abstract

In this study, we applied machine learning tools to automatically track the positions of preschool children in a natural free play setting and derived spatial and temporal features from these data to identify social interactions between them. We observed a sample of 20 preschool children (10 female, 10 male; *M *± *SD *= 3.95 ± 0.82 years) in groups of three children each. Friendship among children was assessed, and friend dyads were paired either with a mutual friend (*n *= 12 groups) or with a mutually disliked peer (*n *= 11 groups). We used a ceiling‐mounted camera to record 10‐min free play sessions of the 23 groups and used an automated keypoint tracking software to extract children's locations over time from the videos. From this data, we derived the following measures for each dyad within the group: distance, social orientation, and paired correlations of children's position and speed. Additionally, a human rater coded all occurrences of social interactions in the videos. Automated measures reliably predicted the occurrence of children's social interactions, validating our choice of spatial and temporal features. Friend dyads were closer, oriented more toward each other, and showed higher position and speed correlations than non‐friends. Social orientation and speed correlation varied over time, and speed correlation increased in mixed‐group contexts, especially among friends. These findings highlight the value of tracking‐based approaches for detecting both fine‐grained interactive behavior and affiliative ties, offering key insights into the spatial dynamics of young children's peer interactions.

## 1 Introduction

Our movement in relation to others both shapes and is shaped by our social relationships (Hall, 1963). We instinctively approach those we like and distance ourselves from those we do not. The ability to regulate physical movement appropriately is crucial for making others feel comfortable around us. We orient towards each other or co‐orient to an object when engaging in social interaction. Moving with others in space and time thus serves a social function, whether intentional—such as in rhythmic activities like dancing (Ehrenreich, 2006)—or spontaneous—such as synchronizing gait while walking (Soczawa‐Stronczyk et al., 2019; van Ulzen et al., 2008), even when instructed not to (Schmidt & O'Brien, 1997). While movement coordination plays a crucial role in shaping social interactions across the lifespan (Rauchbauer & Grosbras, 2020), its developmental origins are particularly interesting for understanding how children begin to navigate their social world.

**Research Highlights**

This study used automated tracking of preschool children's positions and orientation in a natural setting to identify social interactions.

Derived measures of distance, orientation, and coordination of position and speed reliably predicted children's social interactions, validating our choice of spatial and temporal features.

Our measures distinguished friends from non‐friends, with friends being closer, showing more social orientation toward each other, and higher coordination of position and speed.

Automated tracking methods used to derive spatial and temporal features provide valuable insights into early peer social dynamics while minimizing observational interference.

The onset of independent locomotion marks a key developmental milestone that expands children's opportunities for exploration and social interaction, laying the foundation for more complex forms of social behavior (Campos et al. 2000; Goldfield 1995; Piek et al. 2008). Initially, toddlers spontaneously coordinate their movements with caregivers, for example, aligning their locomotion attempts with mothers during free play (Hoch et al. 2021) or adjusting proximity depending on the context (Barnett et al. 2022). With increasing locomotor independence, children begin to actively select other social partners, giving them greater agency in forming their emerging relationships with peers. Peer relationships are characterized by shared competencies and mutual learning, in contrast to the hierarchical structure of adult‐child interactions (Blatchford et al. 2003; Piaget 1932). This symmetry enables children to take active roles in shaping their social interactions (Ashley and Tomasello 1998).

While younger children mostly engage in dyadic interactions (Ladd et al. 1990), by around age four they increasingly participate in larger peer groups (Benenson et al. 1997). Within these groups, children's preferences for particular social partners can evolve into stable friendships, characterized by reciprocity, shared activities, and coordinated, affectively rich interactions (Gifford‐Smith and Brownell 2003; Hay et al. 2004; Howes 2009; Lenz et al. 2021; Youniss 1981). Friends tend to show more cooperation, complex play, and emotional mirroring than non‐friends (Cornejo et al. 2018; Field et al. 1992; Howes 2009; Moore 2009), and spend more time in close proximity to one another (Hartup et al. 1988; McCandless and Marshall 1957; Messinger et al. 2019). These findings suggest that locomotor independence not only expands children's capacity to choose their interaction partners but also facilitates the formation and expression of affiliative bonds.

Crucially, once children engage in larger groups, their interactions are shaped not only by dyadic friendships but also by the collective dynamics of the group (e.g., Horn et al. 2024). As a result, children's behavioral patterns begin to reflect emergent group‐level processes that become increasingly relevant in more complex social environments such as classrooms. Reliably capturing the moment‐to‐moment dynamics of social behavior in groups remains a major methodological challenge for researchers studying early peer interactions (Santos and Vaughn 2018). Traditional approaches use manual coding of video recordings, which is time‐intensive, difficult to scale, and prone to observer bias, particularly when multiple children interact simultaneously. As a result, many studies rely on relatively coarse behavioral categories or short observation windows, limiting the ability to quantify fine‐grained spatial and temporal structure in children's interactions. Developing objective and scalable measures of social interaction therefore represents a key methodological challenge for developmental research.

Recent advances in computer vision and machine learning make it possible to automatically track individuals’ positions and movement in video recordings using a variety of tools (e.g., Loopy, DeepLabCut, OpenPose). These tools provide highly accurate spatial data while substantially reducing the need for manual annotation. However, their potential for studying children's naturalistic peer interactions has only begun to be explored, and it remains unclear whether spatial features derived from automated tracking can serve as valid indicators of social interaction among young children. Horn et al. (2024) recently proposed using spatial proximity, orientation, and interpersonal coordination as key indicators of social connectedness to quantify complex interactions among preschoolers. Leveraging automated acquisition of these three dimensions may enable us to systematically study the spatio‐temporal structure of children's peer interactions in natural settings and test whether movement‐based features can serve as objective indicators of social engagement.

**Spatial proximity** is a fundamental cue for understanding human social behavior. Classic work on proxemics established that interpersonal distance reflects both the level of social engagement and the nature of relationships (Hall 1963, 1965). This framework distinguishes four zones of social distance, ranging from intimate (0–45 cm), personal (45–120 cm), social (120–360 cm), to public (>360 cm), each affording different levels of involvement and communication. While these distance norms fully develop by about age twelve (Aiello and Aiello 1974; Hayduk 1983), already preschool‐aged children demonstrate expectations about interpersonal space. For example, they expect smaller distances between friends than strangers (Paulus 2018) and interpret closeness as a sign of affiliation (Afshordi 2019; Liberman and Shaw 2019). In natural contexts such as preschool classrooms, children tend to maintain close distances to peers with shared positive social bonds (Howes et al. 1988), and frequent proximity predicts mutual social attention, interaction bids, and friendship nominations (Daniel et al. 2013; Santos et al. 2015; Schaefer et al. 2010). Thus, proximity is a marker of affiliation and a predictor of social interaction. While expert observers often use proximity as an interaction index, there is no standardized criterion for inferring social interaction from physical distance (Messinger et al., 2019). Automated tracking methods offer a reliable, data‐driven approach for quantifying interpersonal distances, thus enabling identification of genuine social contact and underlying dynamics of children's social relationships (Messinger et al. 2019).

However, spatial proximity represents only one facet of children's social dynamics, and additional spatial behaviors may further clarify how young peers engage with one another. While proximity reflects opportunities for interaction, it does not necessarily indicate active social engagement. For example, children may interact across a distance, such as when throwing a ball, without being physically close, yet demonstrating **social orientation**. Beyond proximity, body orientation has been proposed as an important structuring element of social interaction. Hall's (1965) sociofugal‐sociopetal axis distinguishes arrangements that discourage versus facilitate interaction, with oblique orientations supporting comfortable engagement without the confrontational nature of direct face‐to‐face positioning. Such spatial configurations provide cues about general engagement and interaction potential (e.g., Cristani et al. 2011; Montanari et al. 2018; Setti et al. 2015). Critically, however, moment‐to‐moment social interaction is more directly governed by where individuals direct their attention. Eye gaze plays a central role in regulating face‐to‐face interactions (Carpenter and Tomasello 2000) and has been established as a measure of social competence in preschool children (van Rijn et al. 2019), particularly in peer interactions (Waters et al. 1983). In contrast, atypical gaze patterns are associated with autism spectrum disorder (Leekam and Ramsden 2006). Although direct eye contact occurs relatively infrequently in naturalistic peer interactions (Arnold et al. 2000), orienting the head toward an interaction partner constitutes a key behavioral indicator of social attention. In fact, evidence suggests that head orientation provides a stronger cue to attentional focus than body orientation (Cooney et al. 2015; Perrett et al. 1992), indicating that head orientation reflects dynamic, moment‐to‐moment attentional alignment between interaction partners. Taken together, these findings underscore the importance of head orientation as a potential key indicator of ongoing social engagement.

Beyond momentary positioning and orientation, the ability to navigate and coordinate movement within a shared space is a key adaptation to the demands of group living, facilitating smoother and more effective social exchanges (Dunbar 2009). From early in life, **interpersonal coordination** is a core component of everyday social interactions and provides valuable insights into social relationships (Brownell 2011; Sebanz et al. 2006; Xu et al. 2020). Importantly, such coordination can emerge intentionally, through collaborative activities, and spontaneously, when children influence one another's behaviors in shared environments (e.g., Fantasia and Delafield‐Butt 2023). Coordination refers to the temporal covariation of behaviors within complex systems and includes phenomena such as synchronization, coupling, entrainment, and alignment (see Hudson et al. 2023, for review). Extensive research has explored various forms of social coordination in early development (e.g., interpersonal synchrony; Feldman 2007), but few studies have examined how young children coordinate spatial social dynamics. Existing evidence suggests that toddlers spontaneously coordinate their locomotor activity with their mothers during free play (Hoch et al. 2021), and preschool children coordinate their spatial positions in natural peer interactions (Messinger et al. 2019). Such coordination may serve as a mechanism for building and maintaining social bonds, consistent with findings that coordinated movements enhance closeness and affiliation among preschool‐ and school‐age children (Rabinowitch and Knafo‐Noam 2015; Tunçgenç and Cohen 2016). These findings underscore the importance of coordination both as a cue of ongoing social interaction and a potential indicator of affiliative relationships. Yet, little is known about how children coordinate their spatial positioning and locomotor activity in peer interactions, highlighting the need for systematic methods that can capture these dynamics.

### 1.1 Present Study

The aim of the present study was to evaluate whether spatial measures derived from automated video tracking can capture meaningful patterns of social interaction among preschool children in naturalistic peer groups of three. Triads constitute the smallest meaningful group beyond dyads, providing an ecologically valid yet analytically manageable configuration that minimizes visual occlusions and computational complexity (Zhou et al. 2005). We used established computer vision tools to extract positional data and focused on testing the validity of three theoretically motivated indicators of social interaction: spatial proximity, social orientation, and interpersonal coordination. First, we examined whether these features predicted moment‐to‐moment social interaction as coded by an expert observer, thereby testing whether spatial features derived from automated tracking can serve as objective proxies for human‐coded interaction. We hypothesized that automated spatial measures would predict manually coded social interaction. Second, to investigate whether automated measures also reflect underlying affiliative ties, we observed children in friend‐only groups (F groups) and mixed groups consisting of a friend dyad and a mutually disliked peer (i.e., non‐friend; NF groups). Based on manual coding of social interaction, we expected that friend dyads would engage in longer social interaction than non‐friend dyads, and that F groups would show overall longer social interaction than NF groups. Building on this behavioral criterion, we further hypothesized that friend dyads would show closer proximity, more mutual orientation, and higher interpersonal coordination than non‐friend dyads. At the group level, dyads in F groups were expected to show overall closer spatial spacing, more cohesive orientation patterns, and higher coordination than in NF groups. Finally, because affiliative behavior may become more focused when a non‐friend is present, we hypothesized that friend dyads within NF groups would display stronger affiliative patterns (i.e., closer proximity, social orientation, higher coordination) than in F groups, where children's attention and engagement may be more diffusely distributed across multiple friends.

## 2 Method

### 2.1 Participants

Participants were recruited and tested in an urban company childcare facility associated with a research institution and media hub in Vienna, Austria. The sample consisted of 20 preschool children (10 female; 10 male) between 3 and 6 years of age (*M *± *SD *= 3.95 ± 0.82 years). All children were typically developing. We did not collect individual data on parents’ socio‐economic status (SES) and education. However, given the recruitment context, families can be assumed to have had middle to high SES. At least one parent from each family had tertiary education with academic orientation (ISCED 6–8). The sample was international. We obtained information about parental country of origin from 12 of the 20 families. Of these, 22 parents came from European countries (Austria *n* = 8; Germany *n* = 7; Belgium *n* = 2; Croatia, Italy, Serbia, Sweden, Switzerland each *n* = 1) and two parents from India.^1^ German was the commonly used language in the facility, and all participating children spoke German well.

### 2.2 Procedure

**FIGURE 1:**
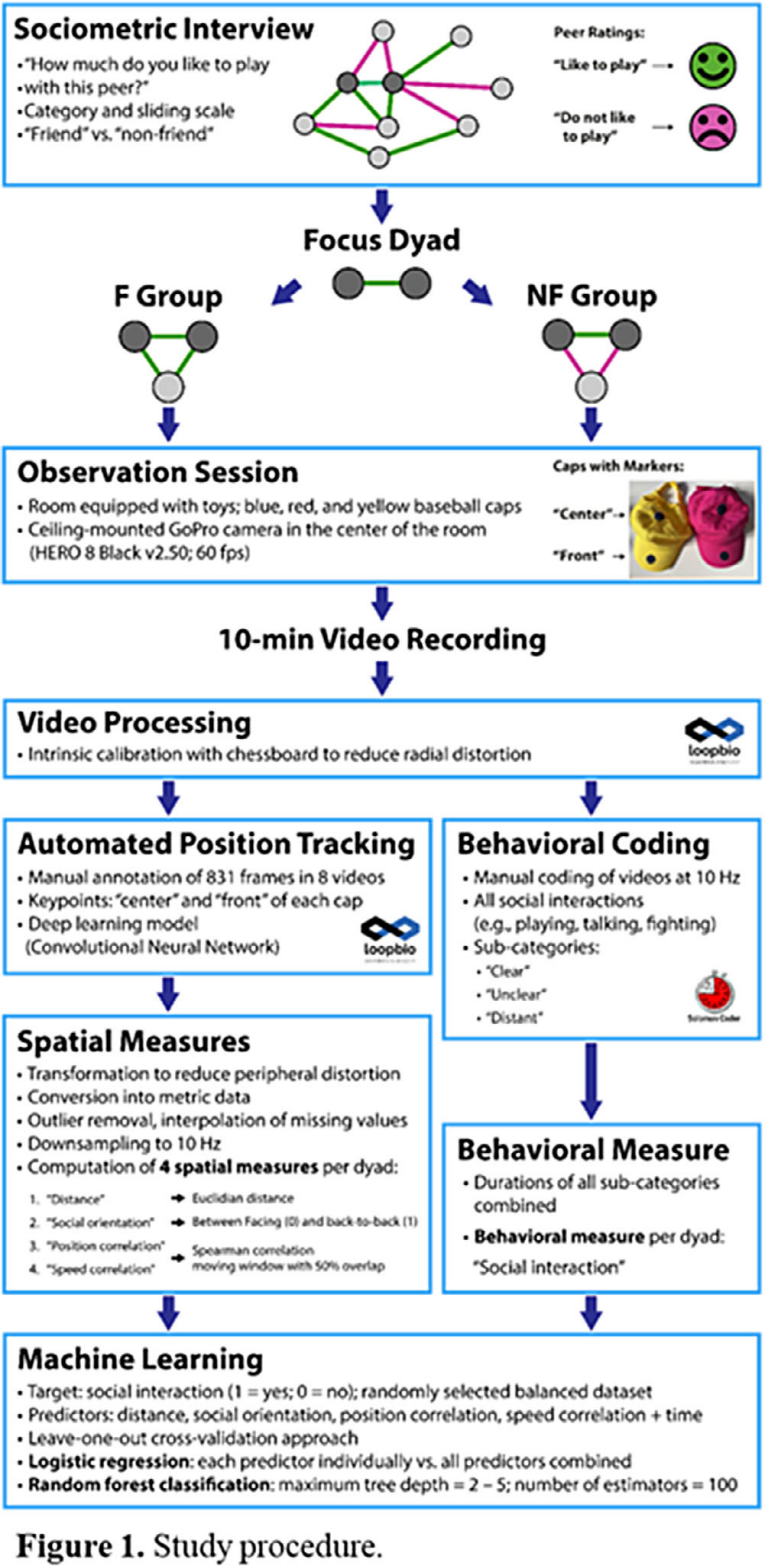
Study procedure.

All parts of the study took place in familiar rooms in the childcare facility among peers that knew each other well. Prior to observation sessions, we used an age‐appropriate sociometric interview to identify friends and disliked peers among the participating children (modified after Birch and Billman 1986; Figure 1). Sociometric interviews were conducted on three test days over a period of 11 days, at a median of 19 days before the observation sessions (*range* = 19–40 days). Each child was tested individually in a separate room for approximately 15 min. To ensure recognition, we first showed the participant photographs of all other participating children and asked them to name each one. The experimenter asked the child to place each peer photograph into one of three sections exemplified by three cartoon faces: (a) happy—“I enjoy playing with this child and I often play with this child,” (b) sad—“I don't enjoy playing with this child and I rarely play with this child,” and (c) neutral—“playing with this child is okay, I don't really care.” For validation, we additionally used a sliding scale with a sad cartoon face on the left and happy cartoon face on the right. The experimenter asked the child to place an arrow along the sliding scale to indicate how much they liked to play with each of the other children. For assessing the degree of liking, we split the sliding scale into three equally sized sections. For both scales, we used ratings in the happy face section as a proxy of being a friend and in the sad face section as a proxy of being a disliked peer, labeled as non‐friend.

Based on data collected via the sociometric interview, we identified 27 friend dyads consisting of two children that had rated each other as friends in both rating scales (gender composition: both female, *n* = 9; both male, *n* = 7; mixed gender, *n* = 11). Eleven of these friend dyads were focus friend dyads, who were tested in two different group constellations: paired once with a mutual non‐friend (NF groups) and at least once with a mutual friend (F groups; Figure 1; seven friend dyads participated in more than one F group). Mutual non‐friends were children who were rated as disliked peers either on both scales by both dyad members (*n* = 7) or in all but one rating from the two dyad members (*n* = 4). One of the focus dyads could only be tested once with a mutual friend, yielding 12 observations where all children were friends (F groups) and 11 observations where one child was a non‐friend (NF groups). In total, we tested 23 groups of three children each (gender composition: all female, *n* = 4; all male, *n* = 2; mixed gender, *n* = 17). Most children were assigned to multiple groups (*Mdn* = 2 groups, min = 1, max = 10).

Group observation sessions took place on 6 test days over a period of 13 days. The order of observations (F, NF groups) was randomized across focus friend dyads. The median interval between two observations of the same focus dyad was 1 day (*range *= 0–9 days). For the observation session, the experimenter brought each group to a familiar room in the preschool facility. Three different rooms were used and room sizes ranged from 2 × 3.5 m to 4 × 6 m (see Figure 3A for an exemplary room). Before the start of the observation, large pieces of furniture were deliberately moved away from the center of the room to create more open space that allowed children to move freely. All rooms were equipped with a range of age‐appropriate toys, with no designated activity areas, allowing children to use all toys freely throughout the space. Apart from the experimenter and the observed children, no other children nor preschool staff were present during the sessions. Each child wore a differently colored baseball cap (red, blue, yellow) with two black marker dots: one in the middle of the front visor (“front”) and one on the crown of the cap (“center”; Figure 1). The baseball caps were used to allow more reliable tracking of the children's orientation via the front and center marker dots and to cover children's faces during video recording and thereby anonymize collected video material. Adherence to cap wearing was high, with brief removals (*Mdn *= 1 s, *range *= 1–8 s) by 16% of all observed children, representing only 0.36% of total observation time. The children were told they could move around the room freely and play with the toys for 10 min. We conducted 10‐min observations to capture representative, natural child behavior, while minimizing fatigue or attentional decline. The experimenter sat quietly in a corner of the room and did not interfere with children's actions. The children were recorded from above using a GoPro camera (HERO 8 Black v2.50; 60 fps) fixed on the ceiling in the center of the room. After 10 min, the experimenter terminated video recording and returned with the children to their classroom.

### 2.3 Automated Position Tracking

Video material was processed using a commercial tracking software, Loopy (http://loopbio.com/loopy/, loopbio GmbH, Vienna, Austria). There are many different options available to track movements from video. Rather than innovate the tracking pipeline, we chose this reliable, off‐the‐shelf solution to obtain accurate positional data for further analysis. To reduce radial distortion from the camera's fisheye optics, we performed an intrinsic calibration by recording a chessboard at various positions, distances and angles, and then applied Loopy's built‐in “undistort video” function to all videos. Next, we used Loopy's supervised machine learning workflow to track the positions of the three children across all frames of each video. An experimenter manually annotated 831 still frames across eight videos. The frames were spaced at even intervals within each video, with the intervals varying across videos (*range* = 50–400 frames), to maximize variability for generalization of the tracking algorithm. In each annotated frame, the experimenter marked the keypoints on each cap (i.e., “front,” “center”) only when clearly visible. Loopy's built‐in convolutional neural network was then trained on these annotations (80% training/validation split; stride 4—meaning the convolutional filter moves in steps of four pixels during feature extraction) to predict the x–y coordinates for all keypoints on each video frame (sampling rate of approximately 60 Hz). Reliability of automated tracking was quantified by comparing the model's predictions for center and front keypoints to a separate set of human‐annotated keypoints in ∼1000 video frames not used in model training. Overall, the difference between human and machine annotations was small: all mean Euclidean distances between the two types of annotations for each keypoint were below ∼2 cm, and all medians were below 1 cm (see Supporting Information for full results).

### 2.4 Computation of Spatial Measures from Positional Data

After the initial lens distortion correction applied in Loopy, we additionally addressed the geometric distortion for objects above floor level created by the overhead perspective by applying a radial transformation function to all x‐y coordinates. In each video, we identified a table for which three points at the base of the legs and three corresponding tabletop corners (i.e., approximately at a child's sitting height) were clearly visible (Figures S2–S4). Using a mouse callback function implemented in *OpenCV* (version 4.10.0; Bradski 2000) in Python (version 3.13.0; Python Software Foundation 2023), we manually selected the x‐y coordinates of these six points and expanded them to create corresponding top and bottom overlay grids. After converting all coordinates to a Cartesian system and aligning the grids by the frame center, we modelled the vectorial difference between corresponding grid nodes (based on radial proximity) using linear regression in *Scikit‐learn* (version 1.6.0; Pedregosa et al. 2011) for each video (see Table S4). Finally, we applied the resulting transformation matrix to all Loopy‐generated x‐y coordinates, producing a consistent inward radial pull that corrected overhead‐perspective distortion across the entire frame.

Using the pixel size of a table‐top in the first frame of each video (for which the size was known in millimetre), we converted the pixel‐based coordinates into metric coordinates and then removed extreme outliers by examining the distribution of the first derivative of each keypoint. Changes exceeding 10,000 mm/s were considered implausible and were replaced with NaN values for center and front keypoints independently. Missing values were then linearly interpolated using SciPy's interp1d function (Virtanen et al. 2020), which also allowed extrapolation of values at the beginning and end of the time series when adjacent valid keypoint coordinates were unavailable. Finally, to match the sampling rate of our behavioral coding (time windows of 0.1 s), we downsampled the data to 10 Hz using SciPy's decimate function, which first applies a low pass anti‐aliasing filter. After preprocessing, all datasets were trimmed to a uniform length of 10 min.

We computed four spatial measures for each possible pair of children within a group: (1) distance, (2) social orientation, (3) position correlation, and (4) speed correlation. ***Distance*** was calculated as the Euclidian distance between a dyad's “center” markers at each time sample. ***Social orientation*** was computed from the head direction inferred from the “front” and “center” markers of each sample. For each child within a dyad, we derived an individual head‐orientation vector extending from their “center” to their “front” marker and computed the angle between this head‐orientation vector and the dyad's “baseline” vector connecting the two children's “center” markers (Lahnakoski et al. 2020). This angle captured the extent to which each child oriented toward their interaction partner. Dyadic social orientation was then calculated as the sum of the two individual orientation angles, yielding a continuous dyadic orientation index ranging from 0° (both children directly facing one another) to 360° (both children fully turned away from one another). Thus, the 0°–360° range reflects the summed orientations of both partners rather than a conventional angular separation measure. To avoid confusion with standard angular measures, these values were rescaled between 0 (face‐to‐face) and 1 (back‐to‐back). Finally, we calculated two correlational measures using a moving window approach, with different windows applied to feature computation for machine‐learning classification (4‐s width used for each sample to capture momentary dynamics) and the analysis of longer‐term temporal dynamics for statistical analysis (1‐min width with 30‐s overlap, as used for time windowing of other automated measures; see details below for more information). ***Position correlations*** were defined as the Spearman correlation between the distance of two children from a fixed reference point (i.e., the top‐left corner of the room)**. *Speed correlations*** were computed as Spearman correlations between the momentary speed (mm/s) of two children, where speed was measured as Euclidean distance between consecutive time samples.

To evaluate whether each spatial measure reflected meaningful structure rather than patterns expected by chance, we constructed a shuffle‐based null distribution for all four measures. For each video, we randomly paired three time series drawn from different videos (without replacement) and recomputed all spatial measures within a 1‐min moving window (with 30‐s overlap). We repeated this procedure 23 times per shuffle set (matching the number of videos) and generated 200 such shuffle sets (total 4600 random combinations). The empirical distributions of these shuffled values served as null models against which real data were compared, and *p* values were computed as the proportion of shuffled values exceeding the observed values in each time bin.

### 2.5 Behavioral Coding of Social Interactions

A primary coder, who was blind to the self‐reported relationships between children, manually coded all videos for social interactions at 10 Hz using Solomon Coder (version beta 19.08.02; András Péter), relying on video only (no audio). The coder was trained through review of sample videos to develop a coding system of children's possible social interactions in the observation setting. One joint coding session with the study team ensured consistent classification of social interactions. Both positive and negative social interactions were included in the coding system (i.e., communicating, playing, fighting; cf. Hay et al. (2021); see Figure S5) and were coded without distinction as “social interaction.” The coded events were further sub‐categorized as “clear” versus “unclear” (based on the coder's confidence in their classification) or “distant” (based on children's distance being more than one arm's length and therefore out of reaching distance). See Supporting Information for details. A second independent observer coded six randomly chosen videos (4 NF groups; 2 F groups; 27% of all videos). To obtain a fine‐grained assessment of reliability, each video was divided into 10 sections of 1 min each and the durations of the social interaction sub‐categories obtained by the two coders were compared with the intraclass correlation coefficient (ICC; two‐way, absolute agreement). Agreement for social interaction across all categories (clear, unclear, distant) was excellent, (ICC = 0.789, *F*(179,180) = 8.50, *p* ≤ 0.001; Cicchetti 1994). Agreement was excellent and good, respectively, when testing interrater agreement individually for the sub‐categories “clear” (ICC = 0.791, *F*(179,122) = 9.06, *p* ≤ 0.001) and “distant” (ICC = 0.707, *F*(179,157) = 5.99, *p* ≤ 0.001). Agreement was poor for the sub‐category “unclear” (ICC = 0.280, *F*(179,141) = 1.86, *p* ≤ 0.001). For all further analyses, we therefore used all instances of social interaction pooled across all three sub‐categories.

### 2.6 Machine Learning

On a sample‐by‐sample basis, we used the four automated measures to predict the manually coded instances of social interaction. We used single sample distance and social orientation measures, while correlations were calculated using 4‐s fully overlapping moving windows (i.e., ±2 s around the sample of interest) to better capture moment‐to‐moment coordination. Both correlation measures were calculated on as small a window size as possible (i.e., 40 samples), balancing sample size needed to estimate correlation coefficients while reducing influence of past and future samples. We considered alternative window sizes to calculate the correlation coefficients (2 s; 10 s; 20 s), which yielded very similar results for the classification task and were not used in the final analysis. Measures and coding both had a sampling rate of 10 Hz and the classification task was performed on each sample independently. Time (sample number) was included as a covariate. To evaluate predictive performance, we used a leave‐one‐out cross‐validation approach in which each video served once as the validation set (Hastie et al. 2009). Because social interactions were less frequent than non‐interactions, the training sets were balanced by including all samples labeled as social interaction (1) and randomly selecting an equal number of non‐interaction samples (0) from across all videos (resulting in 98,800–106,800 samples per category per fold). As baseline models, we fitted logistic regressions using each predictor individually. We then tested a multivariate logistic regression including all predictors simultaneously. Features were *z*‐scored to enable comparison of the fitted weights. Validation sets were left unbalanced regarding social interactions/non‐interactions and were *z*‐scored using parameters derived from the corresponding training data. Additionally, to confirm that the random balancing procedure did not bias our results, we also trained a set of logistic models on the full dataset using balanced class weights, resulting in identical validation performance.

To examine whether interactions among predictors improved performance, we used a random forest classifier approach (Breiman 2001) using the four automated measures and time as predictors. Random forests aggregate predictions from multiple decision trees built from bootstrapped subsets of the data. To fit random forests, we used the Scikit‐learn library (Pedregosa et al. 2011), testing maximum tree depths between 2 and 5, with 100 estimators and default settings for all other parameters. To quantify the importance of the individual predictors, we additionally fitted the multivariate logistic regression separately to each unbalanced, standardized video and assessed the consistency and significance of the resulting coefficients across videos.

### 2.7 Statistical Analyses

Statistical analysis was carried out using R (version 4.5.2; R Core Team 2025) in R studio (version 2024.12.1.563; Posit Team 2025) using packages tidyr (Wickham et al. 2024) and dplyr (Wickham et al. 2023) for data wrangling, and ggplot (Wickham 2016), ggrain (Allen et al. 2021), and patchwork (Pederson 2024) for figure plotting. For descriptive analysis, we averaged the measures calculated from the full 10 min of video across the three dyads within each observation, and created samples split by group composition (F, NF groups) or including all observations (i.e., all videos). For statistical analyses, we were interested in the time course of the measures across the 10‐min observation period. Therefore, we used a 1‐min moving window with 30‐s overlap. Within each window, we computed the average of a dyad's distance and social orientation measures, calculated position and speed correlations across all samples, and quantified the duration of social interactions. This choice was based on our prior experience of how children's social interactions vary across longer observation periods. Use of shorter time windows (30 s; 10 s) did not greatly affect the results. To compare friend dyads when they were observed in F versus NF groups, we used linear mixed models (lme4, Bates et al. 2015; lmerTest, Kuznetsova et al. 2017; model diagnostics assessed with performance, Lüdecke et al. 2021) for each measure collected from friend dyads in the two groups. Models included the predictors group composition (F or NF group) and time bin as fixed effects, and dyad ID and observation as random effects. Correlation coefficients were transformed using Fisher's transform before entering analysis but are plotted as non‐transformed coefficients. To assess the relationship between dyad type (friend and non‐friend) and group composition (F and NF group) and each measure, we also used linear mixed models, including data from all dyads and observations, using predictors group composition, dyad type and time bin as fixed effects and dyad ID and observation as random effects. Note that each dyad ID had only one dyad type and assignment of dyad type to group and observation is unbalanced by design (F groups contain only friend dyads; NF groups contain friends and non‐friends). All LMMs were fit using REML and statistical significance was assessed using Satterthwaite's method (package lmerTest), with adjusted degrees of freedom reported. Confidence intervals for fixed effects were calculated using the bootstrap method of package lme4.

## 3 Results

### 3.1 Descriptive Statistics

Children engaged on average in 13 social interactions (*range* = 0–95) and spent 2.62 min during the 10‐min period in social interactions (*SD* = 2.37; *Mdn* = 2.01; *range* = 0–9.47). Bouts of social interactions were on average 16.64 s long (*SD* = 34.92; *Mdn *= 5.1; *range *= 0.1–493.4). Descriptives for the sub‐categories of social interaction are depicted in Table 1 (see also Figure S6 for a summary of overall social interaction for each dyad across the 1‐min analysis window).

**TABLE 1:** Descriptive statistics for the sub‐categories of social interaction.

Category	Frequency		Overall duration (min)		
	Mdn	Range	M (SD)	Mdn	Range
Clear	5	0–20	1.08 (1.28)	0.57	0–6.01
Unclear	6	0–42	1.06 (1.20)	0.64	0–6.25
Distant	1	0–40	0.48 (1.18)	0.01	0–7.12

**FIGURE 2:**
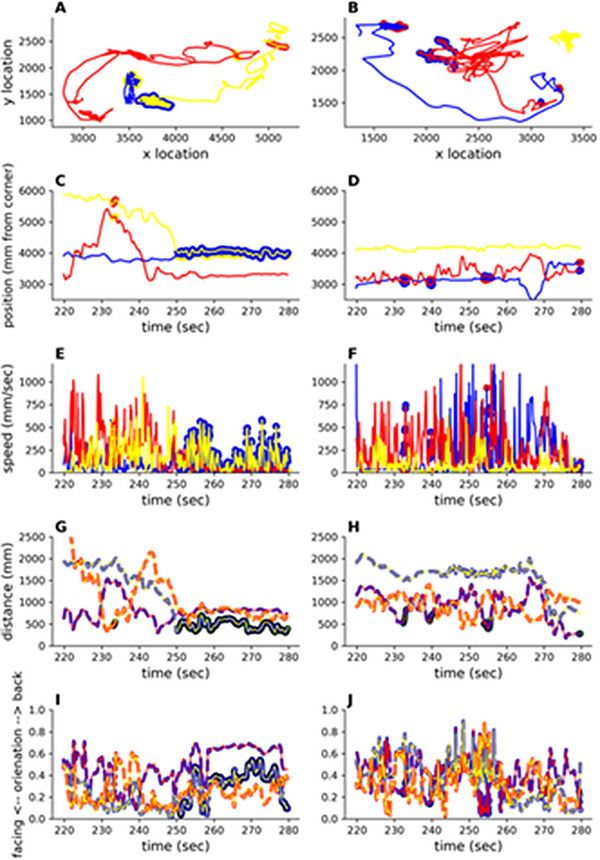
Exemplar plot for two groups of three children wearing different colored hats (red, blue, yellow) in an F group (left column) and an NF group (right column) during a 60‐s segment of the 10‐min free play observation. (A, B) Movement traces as tracked by a computer vision algorithm. (C, D) Position of the children relative to the top‐left corner. (E, F) Speed of the children. (G, H) Distance between pairs of children, represented by color‐coded lines corresponding to the hat colors (e.g., red‐yellow dashed line is the distance between children marked as yellow and red in Panel A or B). (I, J) Social orientation between pairs of children, where 0 indicates children are facing each other, and 1 indicates they are directly facing away from each other. In Panels A–F, manually coded social interactions are marked on each children's trajectory using the color corresponding to their interaction partner (e.g., in Panel C at approximately 235 s, the yellow child interacted with the red child, and vice versa). In Panels G–J, social interactions are marked in black. The same interaction noted above between the red and the yellow child at 235 s is represented by the black mark on the red‐yellow dashed line.

Table 2 displays descriptive statistics for all automated measures and manually coded social interactions (see also Figures S7–S10 for visual summaries of all automated measures for each dyad across the 1‐min analysis window). Figure 2 illustrates the automated measures for a representative group in both the F and NF group. All four automated measures exceeded the shuffle‐based null distribution, indicating that each captured meaningful structure in the children's behavior (see Figures S11–S14).

**TABLE 2:** Descriptive statistics for all measures.

	F Groups	NF Groups	Overall
	*M* (*SD*)	*M* (*SD*)	*M* (*SD*)
Social interaction (s)	189.99 (100.10)	121.42 (74.48)	157.20 (93.58)
Distance (mm)	985.23 (200.42)	951.7 (279.34)	969.12 (248.86)
Social orientation (a.u.)	0.335 (0.032)	0.343 (0.040)	0.339 (0.035)
Position correlation (r)	0.27 (0.19)	0.16 (0.21)	0.22 (0.21)
Speed correlation (r)	0.04 (0.06)	0.07 (0.07)	0.06 (0.07)

*Note*: a.u. = arbitrary units between 0 (face‐to‐face) and 1 (back‐to‐back).

### 3.2 Machine Learning Classification Models

When predicting whether a timepoint showed a social interaction (including pooled clear, unclear, and distant) from automated measures as single predictors in a logistic regression model, most of these contributed above chance to the classification of social interactions (see Table 3). Distance between children emerged as the strongest single predictor. Combining all predictors slightly increased accuracy, and using random forest classification (max depth = 4) further improved performance, suggesting that interactions between the predictors provided additional predictive power.

**TABLE 3:** Machine learning model predicting all social interactions (clear, unclear, and distant combined).

	Training accuracy %	Cross‐validation accuracy %
Distance	71.8 (0.7)	66.8 (7.7)
Social orientation	58.8 (0.2)	57.4 (4.1)
Position correlation	51.3 (0.1)	50.9 (1.3)
Speed correlation	51.8 (0.1)	53.2 (1.9)
Time	50.3 (0.3)	47.8 (6.3)
Overall	73.4 (0.6)	70.4 (6.2)
Overall—Random forest	75.2 (0.5)	74.7 (7.1)

*Note*: Accuracy values represent means, with standard deviations across folds in parentheses.

Examining importance of predictors in the combined model fitted separately for each observation, distance remained the strongest predictor, *β* = –3.15, *SD* = 1.893, *t*(22) = –7.81, *p* < 0.001, with smaller distances increasing the likelihood of social interactions (Figure S15). Social orientation was the next most important predictor, *β* = –0.754, *SD* = 0.357, *t*(22) = –9.91, *p* < 0.001, indicating that children who were oriented more strongly toward each other were more likely to engage in social interactions. Speed correlation was a weaker but still significant predictor, *β* = 0.07, *SD *= 0.119, *t*(22) = 2.77, *p* = 0.0112, with higher correlations increasing the likelihood of social interactions. Finally, position correlation was the weakest predictor, *β* = 0.002, *SD *= 0.133, *t*(22) = 0.07, *p *= 0.9478, and did not meaningfully contribute to the prediction of social interactions. See Supporting Information for a replication of the machine learning analyses using only “clear” instances of social interactions as the outcome variable (Table S5).

### 3.3 Dyadic and Group‐Level Variations in Social Interactions and Automated Measures

**FIGURE 3:**
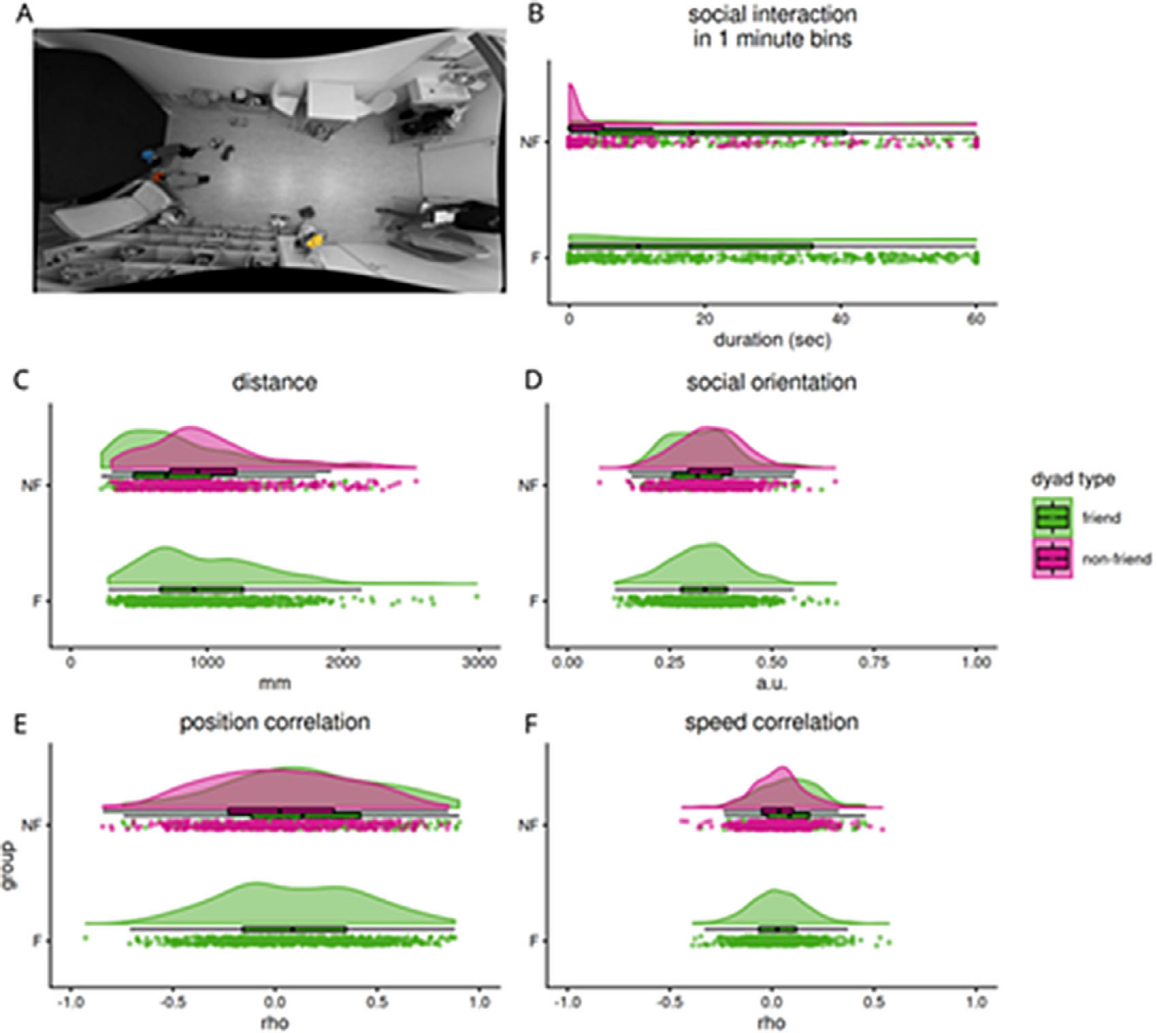
Comparison between friend and non‐friend dyads on the five outcome measures in F and NF groups. (A) Example frame showing a typical testing environment, converted to grayscale except for caps to anonymize children. (B) Durations of manually coded social interactions (second per 1‐min bin) results for all dyads are depicted, with groups (F and NF groups) separated on the y‐axis. Each data point represents the distance of a dyad during a 1‐min time bin of an observation. Where relevant (NF groups), data is divided into dyad type, namely friend dyads (green) and non‐friend dyads (magenta). Raincloud plots are composed of violin plots, boxplots, and datapoints (jittered on y‐axis to aid visibility) providing complementary views of the same samples. (C) Time‐binned distance (mm), details as in B. (D) Social orientation (arbitrary units), where 0 indicates children are facing one another and 1 indicates children facing in opposite directions. (E) Position correlation depicts Spearman correlation coefficients of 1‐min sequences of children's distance from the top‐left of the video. (F) Speed correlation shows Spearman correlation coefficients of the changes in children's positions over consecutive frames in 1‐min sequences.

We examined whether dyadic social interaction and automated measures differed depending on group composition (friend‐only groups, F groups, vs. groups containing a pair of friends and a mutually disliked peer, NF groups), dyad type (friends vs. non‐friends), and time bin (see Tables S6–S9 for full results). For social interaction duration, dyad type had a significant effect, *F*(24.65, 1) = 5.77, *p *= 0.024, with non‐friend dyads interacting for an average of ∼15 s less per 1‐min time bin than friend dyads (*β* = –15.40, 95% CI [–28.16, –4.20]; Figure 3B). No significant effects of group composition, *F*(14.81, 1) = 0.22, *p *= 0.649, or time bin, *F*(1186.19, 1) = 0.13, *p *= 0.722, were observed. For distance, there was a significant effect of dyad type, *F*(34.39, 1) = 12.70, *p *= 0.001, with non‐friend dyads being on average ∼20 cm farther apart than friends (*β* = 202.75, 95% CI [90.24, 333.01]; Figure 3C). Neither group composition, *F*(20.23, 1) = 2.40, *p *= 0.137, nor time bin, *F*(1228.72, 1) = 0.003, *p *= 0.955, had a significant effect on distance. For social orientation, there was a trend for an effect of dyad type, *F*(45.32, 1) = 3.77, *p *= 0.058, with non‐friend dyads showing slightly higher social orientation values (i.e., more turned away) than friend dyads (*β* = 0.023, 95% CI [0.0005, 0.0493]; Figure 3D). Social orientation values also increased slightly but significantly over time bins, *β* = 0.00259, 95% CI [0.00118, 0.00400], *F*(1246.67, 1) = 11.79, *p *< 0.001. For position correlation, dyad type was a significant predictor, *F*(49.45, 1) = 7.57, *p *= 0.008, with non‐friend dyads showing lower correlation coefficients than friends (*β* = –0.243, 95% CI [–0.243, –0.037], Figure 3E). Neither group composition, *F*(27.18, 1) = 1.91, *p *= 0.178, nor time bin, *F*(1246.87, 1) = 0.54, *p *= 0.462, significantly affected position correlation. For speed correlation, dyads in NF groups showed higher correlations than those in F groups (*β* = 0.060, 95% CI [0.030, 0.090], *F*(31.40, 1) = 14.99, *p *< 0.001). Friend dyads had higher correlations than non‐friend dyads (*β* = –0.056, 95% CI [–0.089, –0.029], *F*(63.53, 1) = 12.44, *p *< 0.001, Figure 3F), and correlations increased slightly but significantly over time bins (*β* = 0.00297, 95% CI [0.00025, 0.00565], *F*(1253.77, 1) = 4.73, *p *= 0.030).

### 3.4 Effects of Group Composition on Friend Dyad Dynamics

We examined the effect of social context (i.e., F groups vs. NF groups) on dyadic dynamics among friends using linear mixed models that accounted for repeated measures of friend dyads, co‐occurrences of different dyads within single friend‐only (F) groups, and time‐binned measures within observations (see Tables S11–S15 for full results). For social interaction duration there were no significant effects of group composition, *F*(14.17, 1) = 0.30, *p *= 0.595, or time bin, *F*(507.16, 1) = 1.72, *p *= 0.191. Distance also showed no significant effects of group composition, *F*(14.57, 1) = 1.28, *p* = 0.277, or time bin, *F*(509.83, 1) = 0.12, *p *= 0.726. For social orientation, group composition again had no significant effect on the children's relative orientation, *F*(8.14,1) = 1.0117, *p *= 0.343447, but there was an effect of time bin, *F*(512.74,1) = 8.6149, *p *= 0.003484, indicating a small significant increase in social orientation values (i.e., more turned away) over time (*β* = 0.0035, 95% CI [0.0009731747, 0.005723667], see Table S13; range of possible values is 0–1). Position correlation among friends was not affected by group composition, *F*(17.93, 1) = 0.98, *p* = 0.334, or time (the model had a singular fit due to minimal variance in the random effect of dyad ID). For speed correlation, there was a significant effect of group composition, *F*(10.54, 1) = 17.55, *p *= 0.002, with higher speed correlation observed in friend dyads when grouped with a non‐friend versus another friend (*β* = 0.066, 95% CI [0.036, 0.098]). No effect of time bin was observed for speed correlation, *F*(519.62, 1) = 0.31, *p *= 0.581.

## 4 Discussion

The goal of this study was to identify automated measures that capture preschool children's social interactions with peers in a preschool setting. By combining conventional video‐based coding with position tracking and derived spatial and temporal features, we examined whether spatial proximity, social orientation, and interpersonal coordination could capture not only when children were interacting, but also how friend and non‐friend dyads as well as friend‐only and mixed peer groups differed in their spatial and movement dynamics. Our findings showed that the automated measures reliably predicted children's social interactions and differentiated between friends and non‐friends, corroborating and extending prior research by demonstrating the value of tracking‐based approaches for capturing fine‐grained spatial dynamics of young children's peer interactions and affiliative ties.

We utilized a machine learning‐based motion tracking software (i.e., Loopy) to track children's positions with high spatial and temporal precision. Loopy employs deep learning algorithms to detect user‐defined points of interest in each frame of a video, enabling the automated extraction of spatial coordinates that can be used for later analysis of movement patterns and spatial relationships without the need for time‐intensive manual annotation. From the spatial coordinates, we computed proximity, social orientation, and interpersonal coordination, and validated these features by examining whether they predicted manually coded social interactions. Most automated measures predicted social interaction above chance, with prediction accuracy improving when all predictors and their interactions were modeled jointly. This suggests that children's social engagement is reflected in a constellation of spatial and movement cues rather than any single metric. Importantly, our models were trained and evaluated using human‐coded classifications of social interaction as ground truth. Overall, interrater reliability was good (ICC = 0.789), indicating substantial but not perfect agreement between coders. This level of agreement highlights the inherent difficulty of categorizing social behavior from video and suggests that such annotations are subject to some degree of ambiguity and individual interpretation. Taken together, these findings underscore both the promise of automated approaches for detecting social interactions and the ongoing challenge of establishing solid ground truth data in real‐world peer settings.

Proximity, social orientation, and movement coordination (i.e., speed correlation) all significantly predicted children's social interactions, while co‐occupation of space (i.e., position correlation) did not. Proximity emerged as the strongest predictor, aligning with evidence that physical closeness plays a central role in early childhood interactions, where children rely more on shared space and activities than verbal communication (Gifford‐Smith and Brownell 2003). Being physically close increases opportunities for play, cooperation, and nonverbal exchanges such as shared gaze, gestures, and joint attention, which are foundational elements of early social development (Afshordi 2019; Liberman and Shaw 2019). At the same time, proximity alone does not guarantee that social interaction occurs. In the present study, children remained on average within 1 m of each other throughout the observation, yet only a small proportion of this time was spent in social interaction. This suggests that proximity may be a necessary but not sufficient condition for interaction to occur. Importantly, even within this relatively constrained setting where room size likely limited children's movements, proximity still showed the strongest association with social interaction. This indicates that even small variations in distance carry meaningful information about social engagement. In contrast, co‐occupation of space did not significantly predict interactions, suggesting that simply sharing the same physical area may reflect environmental structure (e.g., classroom layout or activity zones) rather than intentional social engagement (see Messinger et al. 2019).

Beyond proximity, social orientation and movement coordination provided additional insight into how interactions unfold. For example, children frequently oriented toward one another without adopting a fully face‐to‐face position, indicating that early social engagement among peers allows for flexibility between attending to peers and engaging with objects. This is consistent with the idea that interactions among preschoolers are often organized around shared object‐based activities rather than direct face‐to‐face communication (Nasi and Karlsson 2026). Moreover, children appear to move fluidly between moments of coordination and more independent activity. This is consistent with work suggesting that moderate, rather than maximal, coordination is most adaptive for social and cognitive functioning (Gelfand et al. 2020; Mayo and Gordon 2020). Thus, although both orientation and coordinated movement increased the likelihood of social interaction, they may be less indicative of whether interaction occurs, but instead indicate how interaction is structured and maintained over time.

Extending this perspective, the comparatively weaker predictive power of social orientation and interpersonal coordination may reflect a hierarchical organization of social dynamics. Specifically, proximity may define the basic opportunity for interaction, while orientation and coordination capture flexible, moment‐to‐moment processes within this shared space, rather than serving as standalone indicators. Consistent with this interpretation, our classification models that integrated multiple automated measures, particularly those leveraging interactions among predictors, yielded the highest prediction accuracy. This suggests that social engagement among preschool children is best understood as emerging from the interplay of multiple behavioral cues, rather than from any single measure in isolation. Future research could extend this approach by incorporating additional automated measures that capture other dimensions of early social behavior, such as shared object use or gesture dynamics (e.g., Oudah et al. 2020), thereby enabling a more comprehensive characterization of interaction across behavioral channels.

To further test the predictive power of our automated measures, we assessed children's affiliation with their peers and grouped them accordingly into friend‐only and mixed groups. This approach enabled us to compare social dynamics between friend and non‐friend dyads and to explore how these dynamics change with group composition. We found that friend dyads were consistently closer and co‐occupied more similar spatial locations than non‐friend dyads, regardless of whether they were paired with another friend or with a mutually disliked peer. These spatial patterns remained stable over the course of the interaction, corroborating previous findings that young children tend to spend more time near preferred social partners (Howes et al. 1988; Santos et al. 2015). Friend dyads also tended to orient more toward each other and showed higher movement coordination. However, unlike proximity, both orientation and movement coordination fluctuated significantly over the course of the observation, suggesting that they capture short‐term, activity‐driven dynamics rather than stable interactive tendencies. This temporal variability is consistent with our argument that social orientation and movement coordination operate most effectively within moderate and dynamic adjustments that accompany ongoing interactions.

Interestingly, group composition further shaped movement coordination patterns. Mixed groups showed more tightly coordinated movement than friend‐only groups, and friend dyads showed higher movement coordination in mixed than in friend‐only groups. It is possible that in friend‐only groups, children distributed their activities across multiple friends, resulting in more diffuse movement coordination. In mixed groups, the presence of a non‐friend may have strengthened the mutual focus within friend dyads, leading to more reactive and coordinated movement. These dynamics are consistent with findings that friends spend more time playing together (Hartup 1992; Howes 2009; McCandless and Marshall 1957), remain in closer proximity (Altman et al. 2020), and approach each other more readily than dissimilar peers (Banarjee et al. 2023), highlighting the role of peer relationships in shaping children's daily interactions and contributing to the functioning of stable groups (Rubin et al. 2006). These results corroborate our earlier functional distinction between different automated measures, suggesting that distance and co‐occupation of space capture relatively stable aspects of affiliation, whereas social orientation and movement coordination reflect more dynamic, context‐sensitive indicators that track moment‐to‐moment shifts in affiliative interactions.

We used an age‐appropriate sociometric interview to assess children's friendships, although it does not account for the potential instability of young children's friendship ratings. Prior research shows that preschool‐aged children's social preferences are often fluid and context‐dependent rather than reflecting stable, long‐term relationships (Howes 1983). This limitation is particularly relevant, because the test was administered approximately 19 days before the observations took place, leaving ample time for preferences to shift. Moreover, children's ratings may have been influenced by momentary emotions or other factors such as peer gender (e.g., Daniel et al. 2013). Another challenge lies in children's comprehension of the rating scale, particularly when identifying non‐friends. In four cases, a peer was not consistently rated as disliked on both rating scales, limiting the reliability of this category. Future research should incorporate more nuanced assessments of relationship quality and include multiple measurement points to obtain a more comprehensive understanding of children's affiliative ties.

Other limitations of the present study include the relatively small sample size and potential challenges related to both manual and automated measurement approaches. Identifying social interactions from a bird's‐eye view was inherently difficult, as coders relied on macro‐level cues and could not directly observe subtle behaviors (e.g., gaze or affect). As a result, some activities, such as children playing with toys from the same basket, met all coding criteria, yet it remains unclear whether they reflected genuine social interaction. Our automated pipeline also introduced sources of measurement uncertainty. While automated tracking using Loopy provided a feasible and minimally supervised approach to quantify spatial and movement features, peripheral distortion and biases in head orientation detection (i.e., misclassification of the back of the head as the front marker) introduced some error. These limitations indicate a broader trade‐off between scalability and precision. Reducing human involvement increases efficiency and ecological validity, but may also introduce noise at different stages of the process. Accordingly, the level of human oversight needs to be carefully considered across tracking, feature extraction, and analysis. Despite these constraints, our relatively simple, snapshot‐based approach was able to generate meaningful predictions of human‐coded social interactions. Addressing these limitations may involve incorporating child‐specific models and multi‐camera setups, which could reduce tracking ambiguities and enable richer feature extraction.

Future research could further explore automated measures to capture additional behavioral cues, such as velocity of approach and withdrawal (e.g., Banarjee et al. 2023) or synchronized locomotion (e.g., Heravi et al. 2018), as well as finer‐grained measures such as social gaze or affect. Combining these with simultaneous recordings of vocalizations (e.g., Altman et al. 2020; Dai et al. 2020; Irvin et al. 2021) and tracking of elements such as toys, hand position, and full body or shoulder orientation could further enrich the analysis of interactive behaviors. In this context, sensing technologies, including markerless tracking systems and wearable devices, offer valuable tools for capturing real‐time, multimodal data, enabling a more nuanced analysis of social patterns and relationships (Foster et al. 2024; Horn et al. 2024). These tools also enable studies of larger peer groups, collective behavior, and group‐level phenomena (e.g., F‐formation patterns; Cristani et al. 2011), expanding the scope and generalizability of automated assessments in early childhood settings.

The present study demonstrates that social interactions among preschool children can be predicted with high accuracy using minimal equipment, highlighting the feasibility of applying computational approaches in naturalistic settings with low logistical demands. Such methods should always be carefully and thoughtfully applied (see de Barbaro et al. 2026), yet they hold considerable potential for addressing complex questions regarding early peer social interactions. Given that preschool peer groups consist of individuals with unique traits, dyads with specific relational histories, and dynamic subgroups embedded within larger networks, a multi‐layered analytical framework is necessary to capture these complex social dynamics (Horn et al. 2024). Automated tools also offer valuable insights into atypical developmental trajectories by providing objective, high‐resolution assessments of motor and social behaviors in natural settings (Ossmy et al. 2025). Advanced machine learning methods, paired with richer behavioral data, will enable increasingly precise classification of both the form and quality of early peer interactions, offering deeper insights into the mechanisms that shape young children's social development.

## Author Contributions

**Gabriela Markova**: conceptualization, methodology, supervision, funding acquisition, project administration, writing – original draft, writing – review and editing. **Jozsef Arato**: data curation, formal analysis, writing ‐ original draft, writing ‐ review and editing, software, visualization. **Ruzena Ceral**: data curation, investigation, formal analysis, writing ‐ original draft, writing ‐ review and editing, visualization. **Maximilian Hofbauer**: software, resources, writing – review and editing. **Cliodhna Quigley**: data curation, formal analysis, writing – original draft, writing – review and editing, software, visualization. **Lisa Horn**: conceptualization, methodology, data curation, supervision, funding acquisition, project administration, writing – original draft, writing – review and editing.

## Conflicts of Interest

The authors declare no conflicts of interest.

## Ethics Statement

All procedures were carried out in accordance with the Declaration of Helsinki and had been approved by the ethics committee of the University of Vienna (Ref. No. 00599). Prior to the study, informed consent was obtained from all participants’ parents or legal guardians and the children's participation in the study was voluntary.

## Supporting information




**Supporting file**: desc70241‐supp‐0001‐SuppMat.pdf

## Data Availability

Processed data and scripts are publicly availableat https://doi.org/10.17605/OSF.IO/VEKAB.

## Endnotes

1 Since ethnicity is not collected or commonly used in Austrian population statistics, we instead report parents’ self‐reported countries of origin.
