# Supplementary material for: Decoding Preschool Social Dynamics: Automated Tracking of Spatial and Temporal Patterns to Investigate Social Interactions and Relationships in Peer Groups
Source: Dev Sci. 2026 Jun 18;29(4):e70241. doi: 10.1111/desc.70241 (PMC13278523; doi:10.1111/desc.70241)
Supplement: Supplementary file 1 — Supporting file: desc70241‐supp‐0001‐SuppMat.pdf [file DESC-29-e70241-s001.pdf]

Supplementary Material

Validation of automated tracking

To validate the results of the automated tracking results, a human coder manually annotated a total of 1003 frames from 4 videos, using regular spacing of frames in each video so that a variety of positions, postures, and lighting conditions were included. None of these videos were used in training the deep learning model used for automated tracking. To quantify the difference between human and machine locations, we first converted pixel values to mm using the transformations detailed in the first two paragraphs of methods section 'Computation of spatial measures from positional data' and then calculated the Euclidean distance between matched keypoint and frame combinations (n = 4,563 datapoints). In the majority of cases, these distances were small (Figure S1, Table S1). We did not plot values above a distance of 10 cm and provide the counts of these occurrences in Table S2. Finally, for both human and machine data, some frames did not have annotations for some keypoints (i.e., the keypoint was occluded) and the number of these occurrences are provided in Table S3.

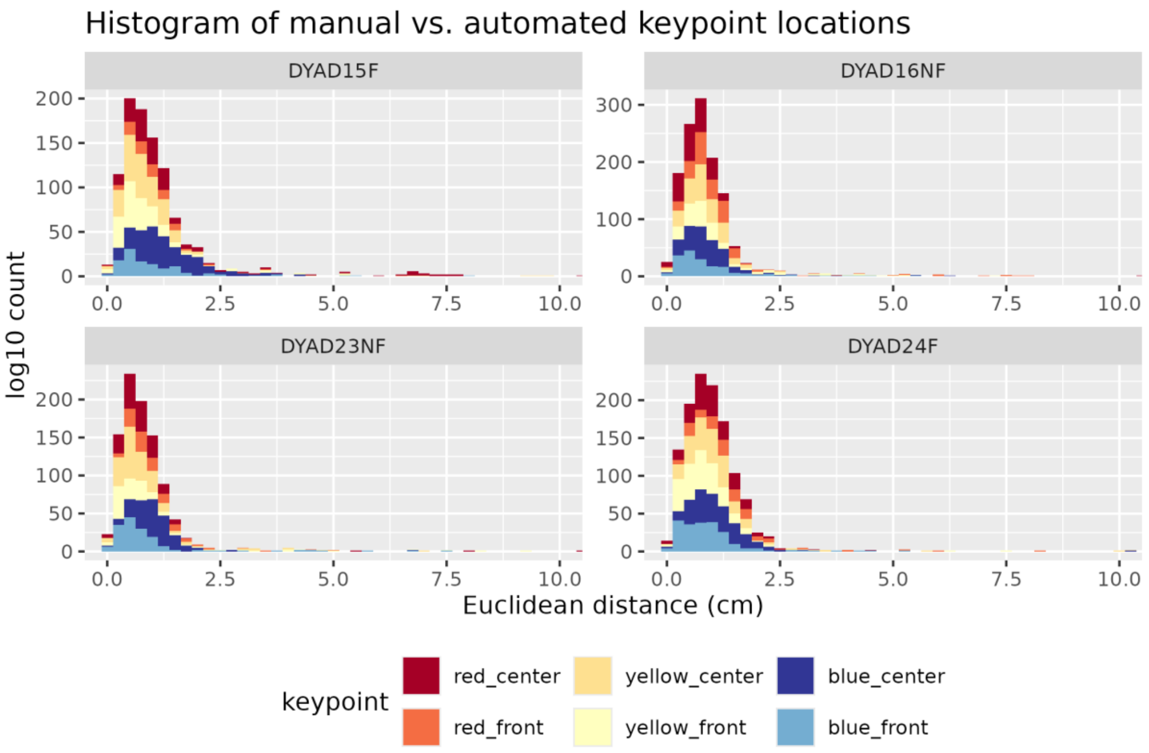

Figure S1. Results of Loopy validation, comparing human with machine keypoint annotations. Each panel depicts the results for one video (title of the respective panel), with colours in the stacked histogram depicting the keypoint used. X-axis is limited to 10 cm to improve visibility.

Table S1. Loopy validation statistics. Mean and standard deviation and median of the Euclidean distance in cm between ~1000 human and machine annotations are shown for each keypoint.

Keypoint	*M* (*SD*)	*Mdn*
Red center	1.07 (1.47)	0.77
Red front	1.85 (9.45)	0.97
Yellow center	1.07 (1.63)	0.79
Yellow front	2.01 (7.24)	0.73
Blue center	1.30 (1.81)	0.99
Blue front	1.09 (1.91)	0.69

Table S2. Number of larger discrepancies in human vs. machine keypoint locations. For each video and keypoint, we list the number of frames for which the distance between human and machine locations exceeded the limits of Figure S1 (>25 pixels).

Video	Red center	Red front	Yellow center	Yellow front	Blue center	Blue front	Sum
DYAD15F	1	2	1	2	6	4	16
DYAD16NF	2	1	0	1	0	1	5
DYAD23NF	3	1	0	21	0	0	25
DYAD24F	1	0	12	1	8	2	24
Total	7	4	13	25	14	7	70

Table S3. Number of missing keypoint occurrences. The total number of frames (out of 1003) is listed by keypoint and annotation type for which that keypoint was not obtained.

	Blue center	Blue front	Red center	Red front	Yellow center	Yellow front
Automated	96	284	202	426	101	114
Manual	38	156	90	318	13	157

Figures illustrating the geometric distortion correction

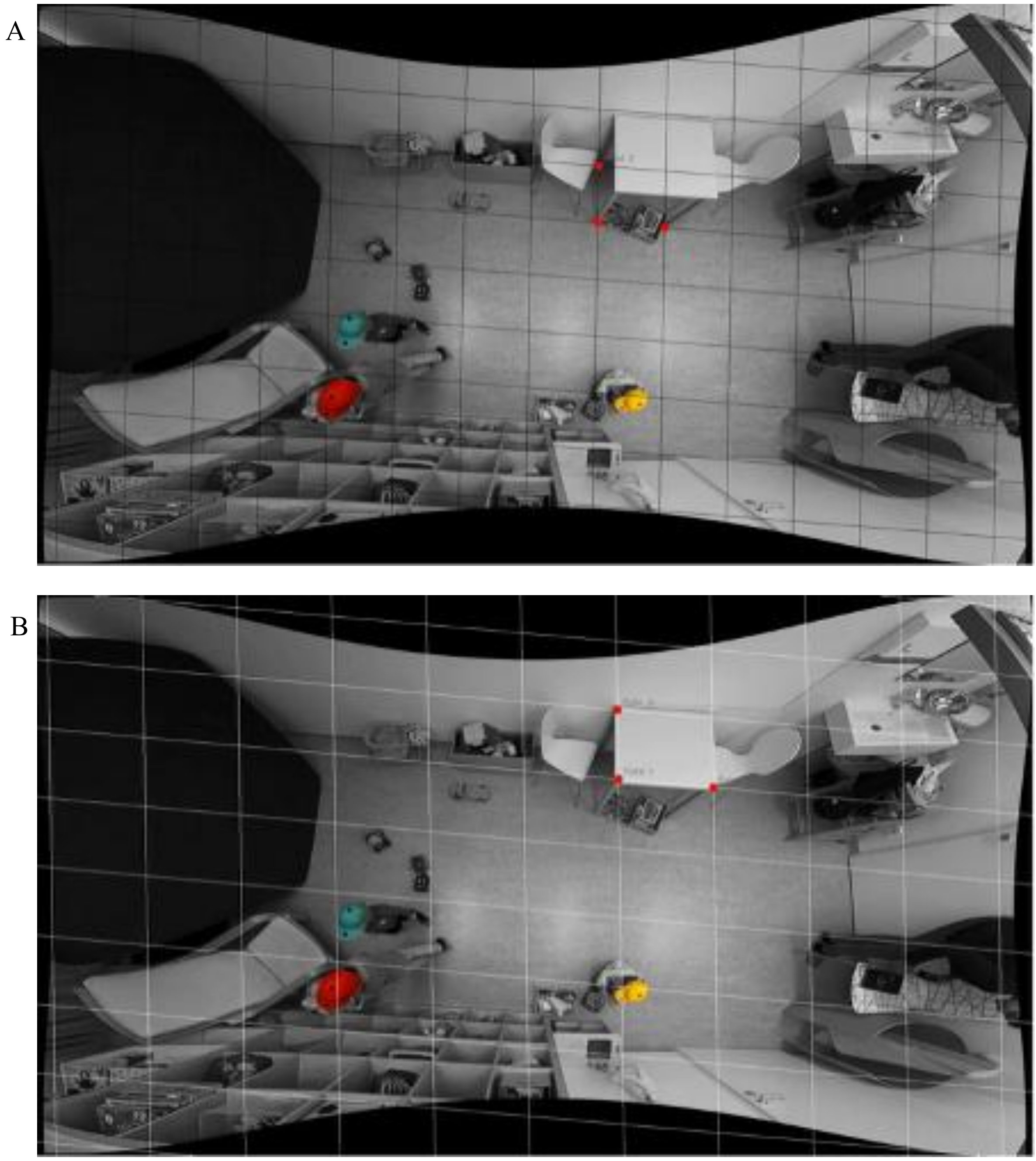

Figure S2. Bottom and top grid derived from hand-selected points. A. Hand-selected points at the base of table legs (red) serve as nodes of the bottom overlay grid (black). B. Hand-selected points at the tabletop (red) serve es nodes of the top overlay grid (white).

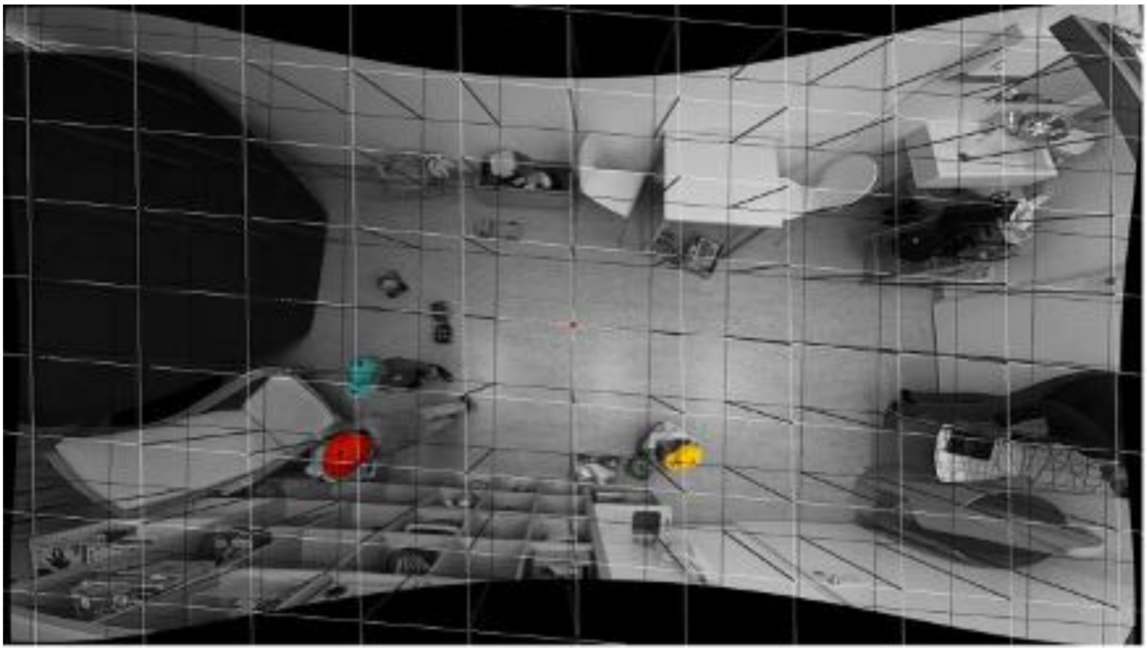

Figure S3. Transformation computed from vectorial grid nodes difference. The top and bottom grid were aligned by the center of the frame and vectorial distance between corresponding nodes has been computed (depicted as radial thick lines connecting grid nodes, red in the middle).

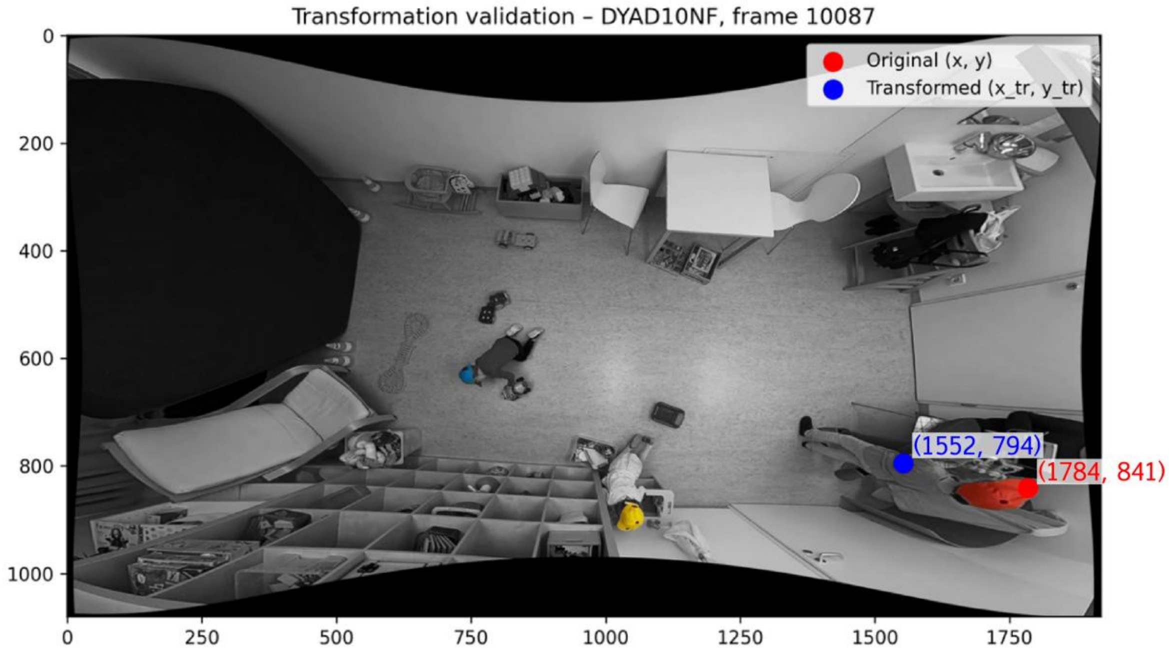

Figure S4. Visual validation check for the direction of the radial pull. In this example, the right-most datapoint ("front" marker of red cap) located at x = 1784.20, y = 841.12 (red) was transformed to x_t = 1552.43, y_t = 794.28 (blue). This transformation represents a radial pull at the level of the top grid node in the direction to the level of the bottom grid node. The coordinates were overlaid over the corresponding frame number 10087 with the right-most datapoint in the video. The x-axis represents the x-coordinates of the standard top-left-origin (non-Cartesian) screen space, and the y-axis represents the corresponding y-coordinates in that same system.

Linear radial distortion formulas

Table S4. Linear coefficients (intercept, slope) derived from differences of nodes between two grids. Coefficients were estimated per video observation and applied to Cartesian coordinates as follows: $x_t = a * x + b * y$ and $y_t = c * x + d * y$, where a and b are the linear coefficients for transformation of the x-coordinates, and c and d for the y-coordinates. The variables x and y represent the original coordinates before transformation, while the variables x_t and y_t represent the corresponding transformed coordinates.

Video observation	Formula derived for x-coordinates	Formula derived for y-coordinates
DYAD02F	$x_t = 0.8639 * x - 0.0154 * y$	$y_t = - 0.0023 * x + 0.9953 * y$
DYAD06F	$x_t = 0.5881 * x - 0.0204 * y$	$y_t = - 0.0028 * x + 0.6999 * y$
DYAD06NF	$x_t = 0.6016 * x - 0.0021 * y$	$y_t = - 0.0041 * x + 0.7009 * y$
DYAD10F	$x_t = 0.7280 * x - 0.0025 * y$	$y_t = - 0.0054 * x + 0.8282 * y$
DYAD10NF	$x_t = 0.7069 * x - 0.0326 * y$	$y_t = - 0.0090 * x + 0.8199 * y$
DYAD11F	$x_t = 0.6960 * x + 0.0230 * y$	$y_t = - 0.0025 * x + 0.7516 * y$
DYAD11NF	$x_t = 0.7118 * x - 0.0157 * y$	$y_t = - 0.0023 * x + 0.7550 * y$
DYAD12F	$x_t = 0.7494 * x + 0.0299 * y$	$y_t = - 0.0149 * x + 0.8174 * y$
DYAD12NF	$x_t = 0.7164 * x + 0.0052 * y$	$y_t = - 0.0025 * x + 0.7733 * y$
DYAD14F	$x_t = 0.7392 * x - 0.0106 * y$	$y_t = - 0.0033 * x + 0.7882 * y$
DYAD14NF	$x_t = 0.7178 * x - 0.0145 * y$	$y_t = - 0.0100 * x + 0.8203 * y$
DYAD15F	$x_t = 0.7196 * x - 0.0182 * y$	$y_t = - 0.0067 * x + 0.8424 * y$
DYAD15NF	$x_t = 0.7285 * x - 0.0011 * y$	$y_t = - 0.0021 * x + 0.7762 * y$
DYAD16F	$x_t = 0.7278 * x - 0.0051 * y$	$y_t = - 0.0052 * x + 0.7955 * y$
DYAD16NF	$x_t = 0.7007 * x - 0.0099 * y$	$y_t = - 0.0035 * x + 0.7777 * y$
DYAD18F	$x_t = 0.7291 * x + 0.0020 * y$	$y_t = - 0.0056 * x + 0.8502 * y$
DYAD18NF	$x_t = 0.7207 * x - 0.0054 * y$	$y_t = - 0.0060 * x + 0.7985 * y$
DYAD21F	$x_t = 0.7206 * x + 0.0078 * y$	$y_t = - 0.0057 * x + 0.8253 * y$
DYAD21NF	$x_t = 0.7169 * x - 0.0270 * y$	$y_t = - 0.0064 * x + 0.8332 * y$
DYAD23F	$x_t = 0.7407 * x - 0.0232 * y$	$y_t = - 0.0021 * x + 0.7704 * y$
DYAD23NF	$x_t = 0.7179 * x + 0.0203 * y$	$y_t = - 0.0023 * x + 0.7682 * y$
DYAD24F	$x_t = 0.7866 * x + 0.0194 * y$	$y_t = - 0.0129 * x + 0.8342 * y$
DYAD24NF	$x_t = 0.6972 * x + 0.0129 * y$	$y_t = - 0.0100 * x + 0.7864 * y$

Coding system for the manual coding of social interactions

Both positive and negative social interactions were included in the coding system (i.e., communicating, playing, fighting; cf. Hay et al. 2021) and were coded – without distinction – as “social interaction”. The coded events were further sub-categorized as “clear” vs. “unclear” (based on coder's confidence in their classification) or “distant” (based on children's distance being more than one arm's length and therefore out of reaching distance). If children showed a behavior described in the coding system within reaching distance of each other and engaged in body/head orientation towards one another at least once within a 10-s interval or touched the same object, this was categorized as “clear”. If the body/head orientation criterion was not met and/or the coder was uncertain whether a behavior defined as a social interaction in the coding system occurred, this was categorized as “unclear”. If a social interaction occurred outside of reaching distance (e.g., throwing an object to each other over a distance), this was categorized as “distant”.

CODE "SOCIAL INTERACTION" WHENEVER CHILDREN "COMMUNICATE", "PLAY", AND/OR "FIGHT"

CODE "CLEAR" WHEN YOU ARE SURE THAT ALL PRECONDITIONS ARE MET

CODE "UNCLEAR" WHEN YOU ARE NOT SURE THAT ALL PRECONDITIONS ARE MET

Act	Precondition	Behaviour	Cues
communication	reaching distance (arm's length) plus body and/or head orientation towards each other	gesticulating	pointing (if other's gaze/head movement follows)
			showing palm to demand objected (if answered)
			(both) perfroming the same gesture at the same time
		talking	mouth movement (in turn taking manner)
play	reaching distance (arm's length)	manipulation of joint objects	taking objects from the same basket
			both holding up an object in close proximity
			joint project using different toys (e.g. building a train track)
			performing similar actions with similar objects
			offering and rejecting objects
		bodily play	turn-taking in imitating movements
		exchange of the same object	turn-taking manipulation of the same object
physical fight	physical contact	bodily aggression	kicking an intruder away
			hitting the other, even one-sided
fight over resources	reaching distance (arm's length)	manipulation of other's object	kicking, pushing or throwing other's toy away
			destroying other's toy
			taking object away from the other

CODE "DISTANT SOCIAL INTERACTION" WHENEVER CHILDREN INTERACT AT DISTANCES FURTHER THAN REACHING DISTANCE

Act	Precondition	Behaviour	Cues
distant interaction (various)	outside reaching distance (arm's length) plus body and/or head orientation towards each other	communicating across the room	mouth movement and/or gestures directed at each other (in turn taking manner)
		exchange of the same object	turn-taking manipulation of the same object (e.g. throwing it back and forth)

Figure S5. Coding system. The coding system detailed the preconditions, behaviors, and cues used by the coder for identifying various instances of social interaction. All instances were coded – without distinction – as “social interaction”.

Figures depicting time-windowed measures

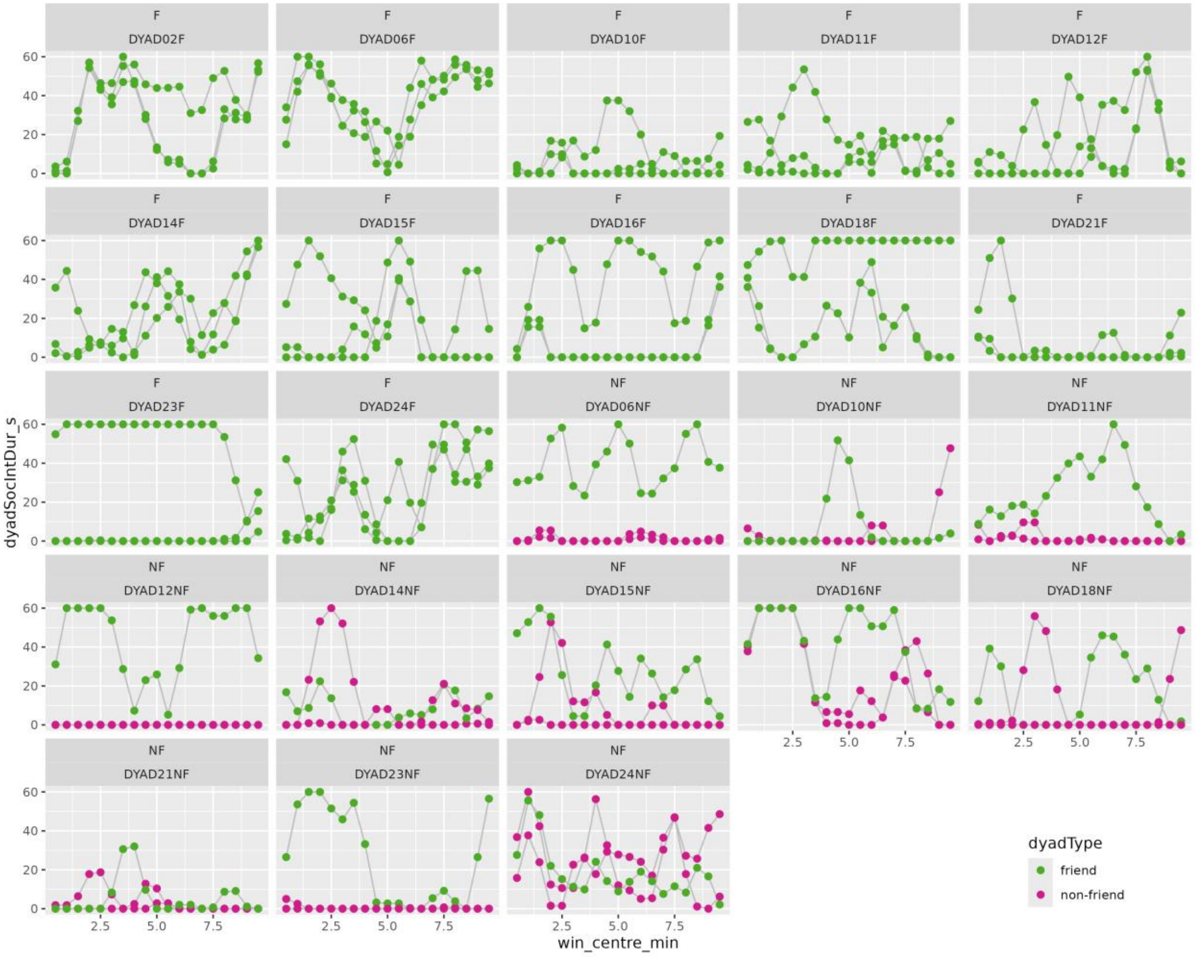

Figure S6. Duration of social interaction. Each panel depicts data from a different video, with each datapoint showing the duration for which that dyad was judged by a trained human coder to be engaging in a social interaction (see Methods). As the data is binned into 1-minute intervals, the values can range between 0 and 60 seconds. Window centers in minutes are depicted on the x axis. Colour indicates friendship status of a dyad (pink: non-friends; green: friends). Data from a single dyad is joined by grey lines. Group composition is indicated in the text above each panel (F: group contains only friends, i.e., 3 friend dyads; NF: group contains two friends, i.e., 1 friend dyad, and one child who neither of the friend dyad children consider to be a friend, i.e., 2 non-friend dyads).

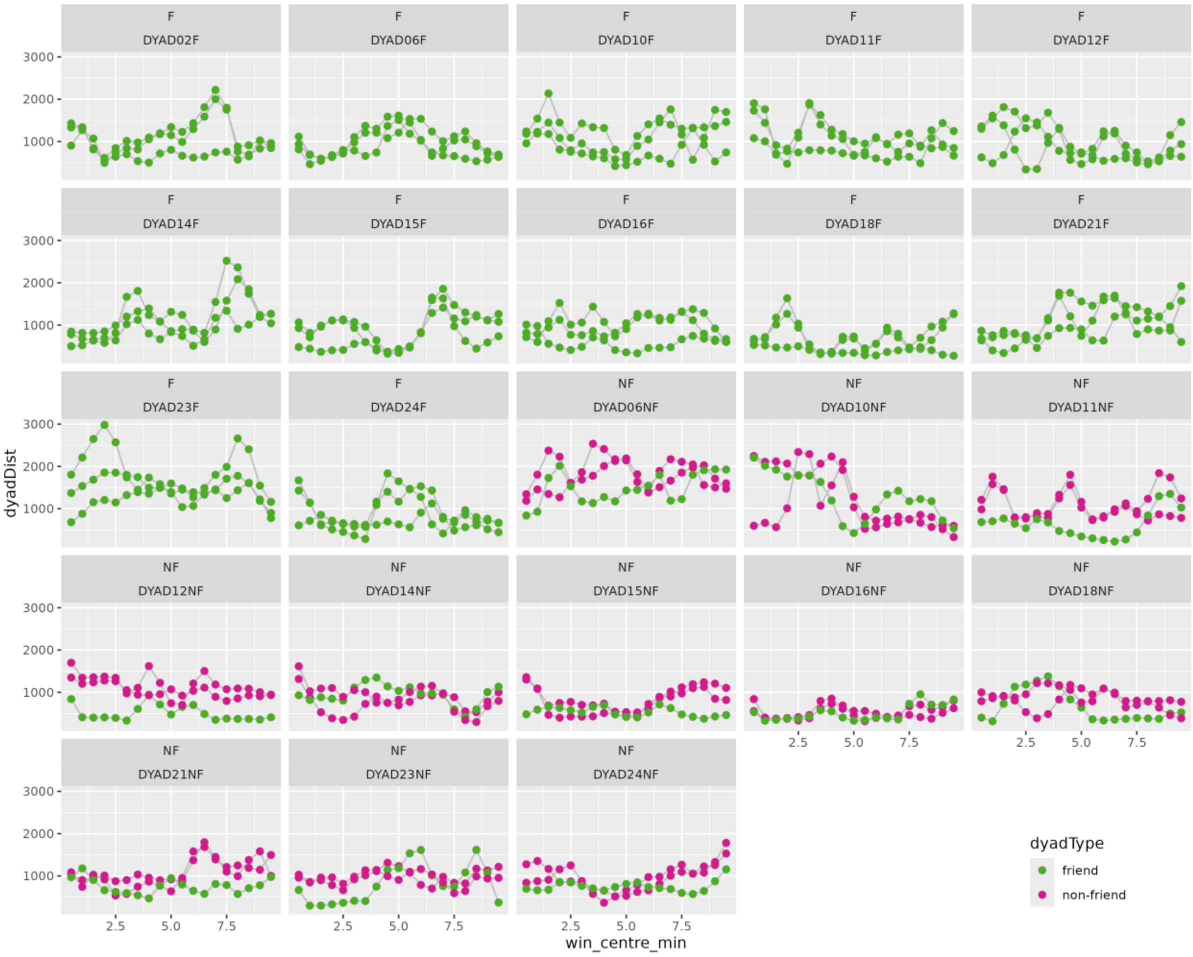

Figure S7. Within-dyad distances. Data is depicted as in Figure S5. Here, each datapoint depicts the average distance in mm between children of a specific dyad during a 1-minute analysis window.

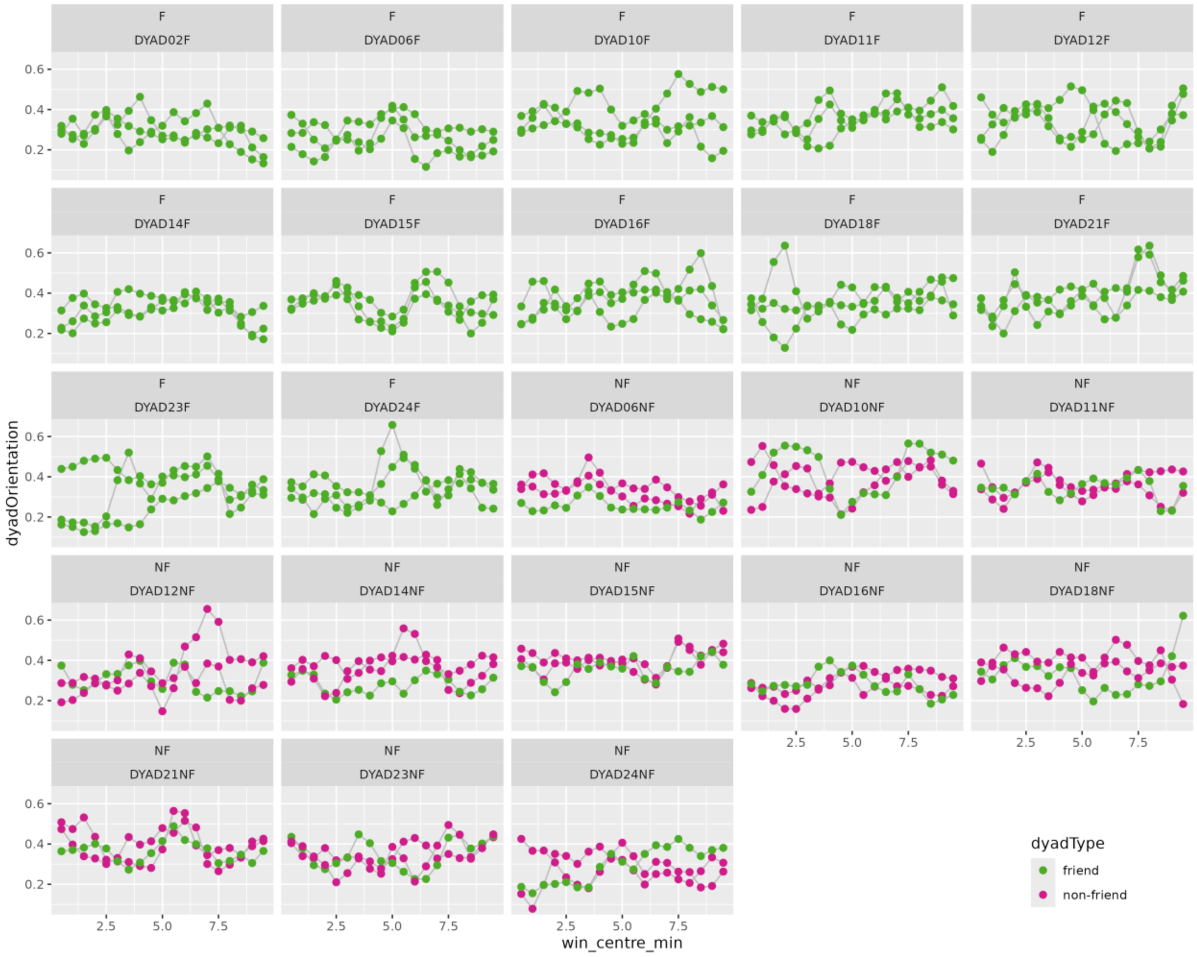

Figure S8. Social orientation of dyads. Data is depicted as in Figure S5. Here, each datapoint depicts the social orientation value of a dyad, which ranges between 0 (face-to-face) and 1 (back-to-back).

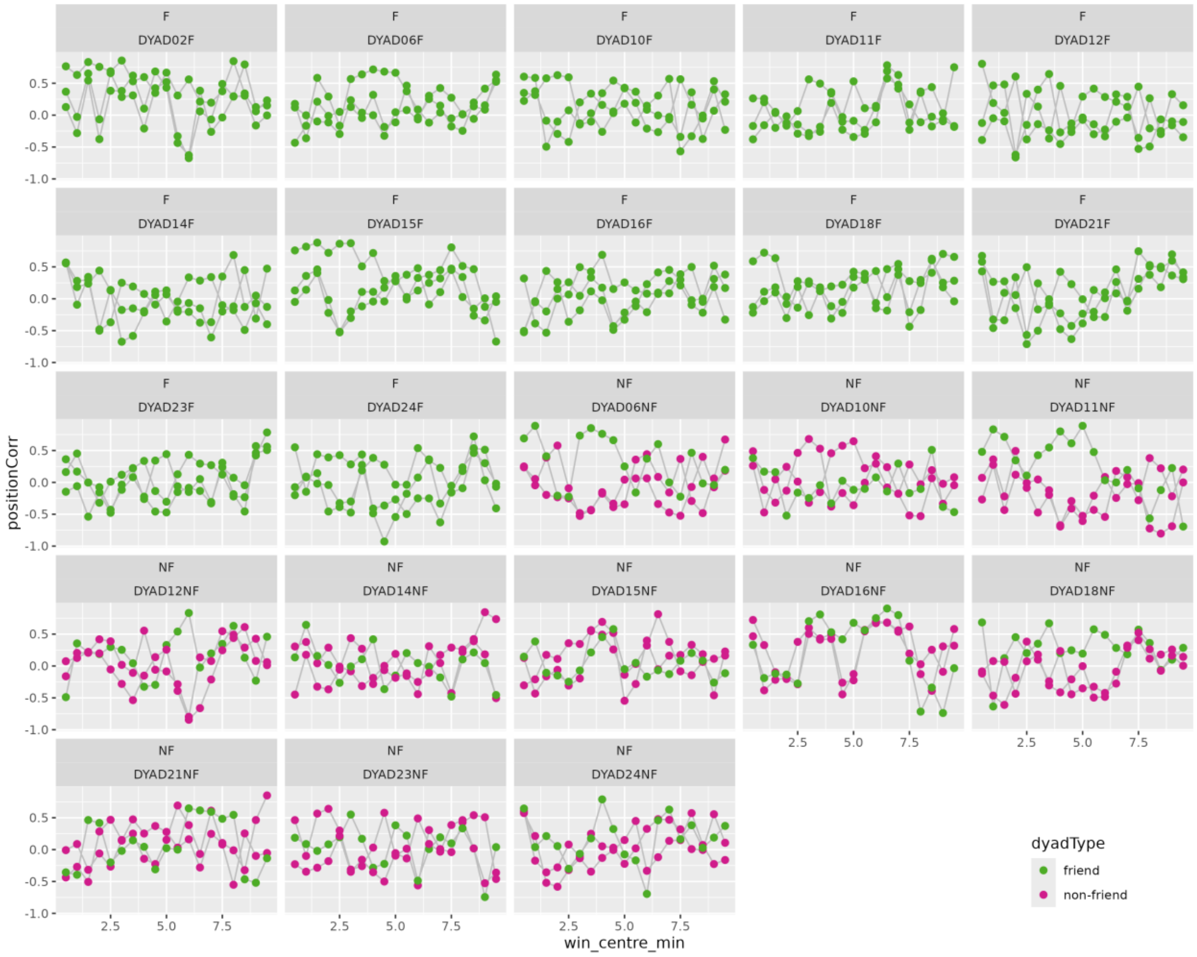

Figure S9. Dyadic position correlation. Data is depicted as in Figure S5. Here, each datapoint depicts the Spearman correlation coefficient of the positions of the two children in a dyad within a 1-minute time bin, with each position measured as a distance from an arbitrary point in the room (top-left corner) to allow for correlation between two unidimensional values.

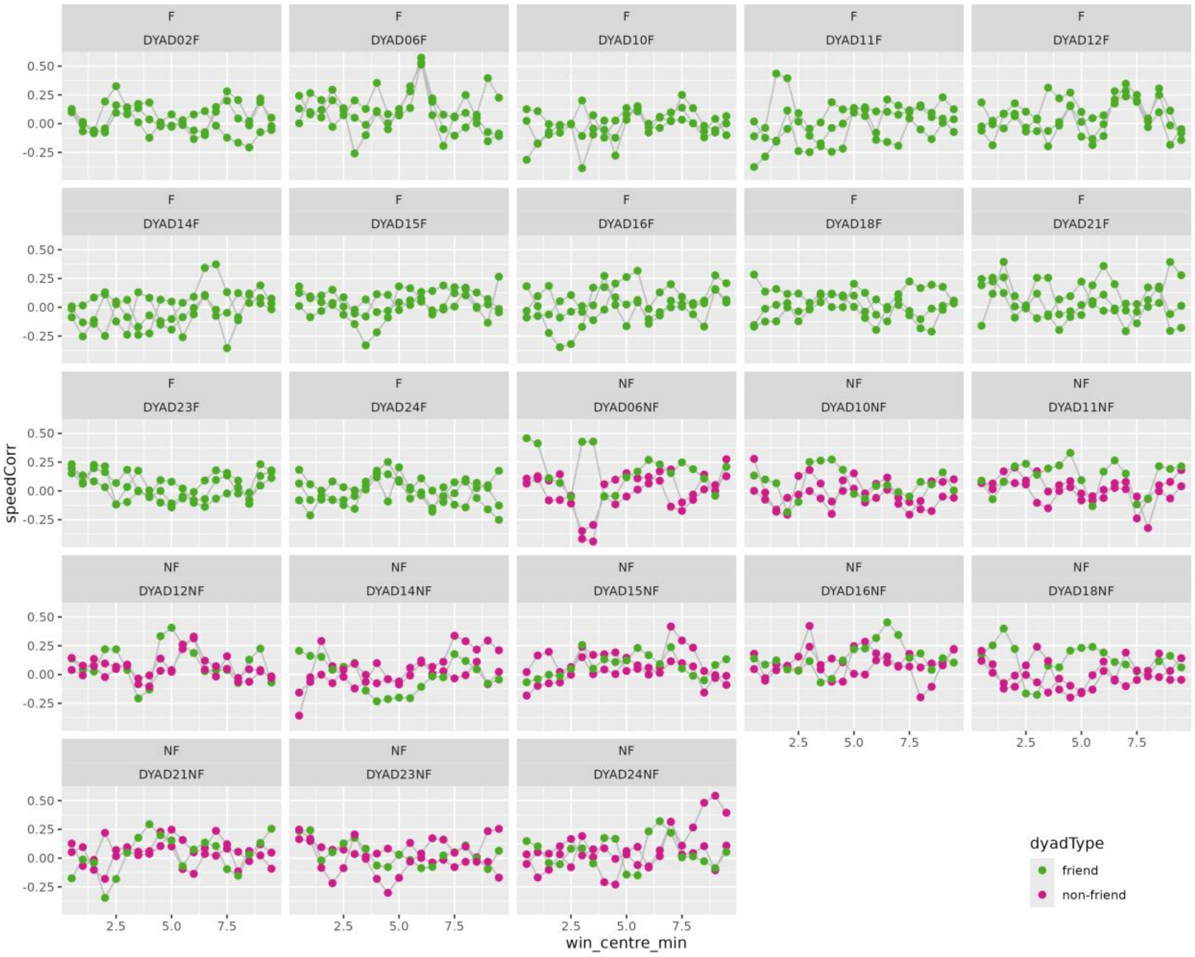

Figure S10. Dyadic speed correlation. Data is depicted as in Figure S5. Here, each datapoint depicts the Spearman correlation coefficient of the speeds of the two children in a dyad within a 1-minute time bin, where speed is measured as Euclidean distance between two consecutive time-points in the time-series of a single child's position data.

Comparison to shuffle-based null distribution for measures

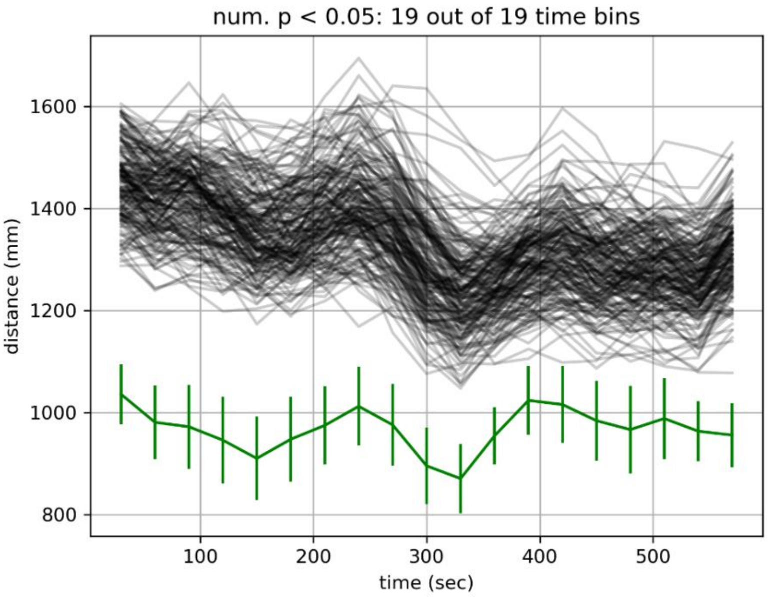

Figure S11. Moving window distance between the children in our data (green) vs. shuffled distribution (black lines) of 200*23 random time series combinations of 3 children from three different videos. All bins show that true distance is smaller than chance.

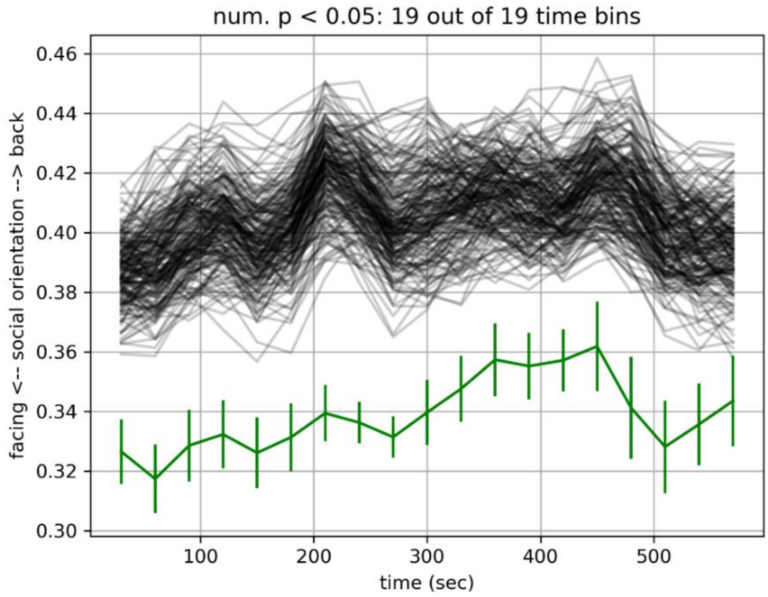

Figure S12. Moving window social orientation between the children in our data (green) vs. shuffled distribution (black lines) of 200*23 random time series combinations of 3 children from three different videos. All bins show that true social orientation values are smaller than chance.

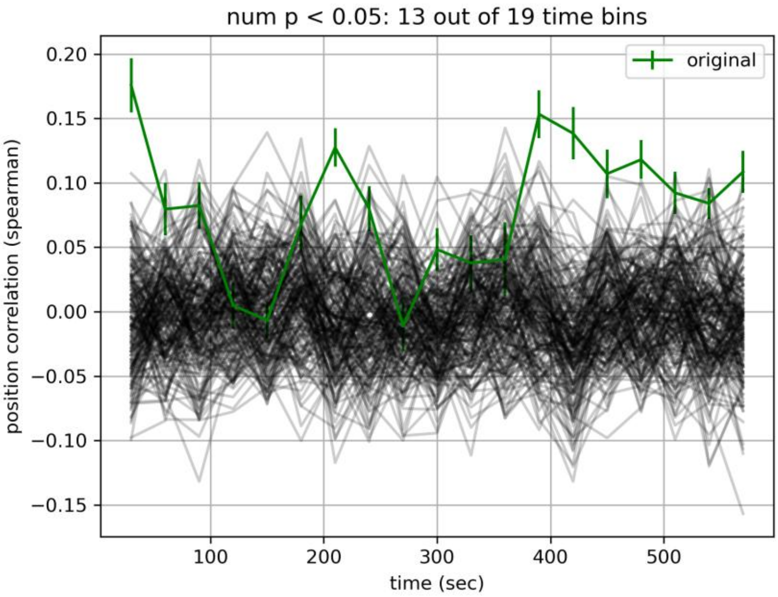

Figure S13. Moving window position correlation between the children in our data (green) vs. shuffled distribution (black lines) of 200*23 random time series combinations of 3 children from three different videos. Thirteen out of 19 time-bins show an above chance position correlation.

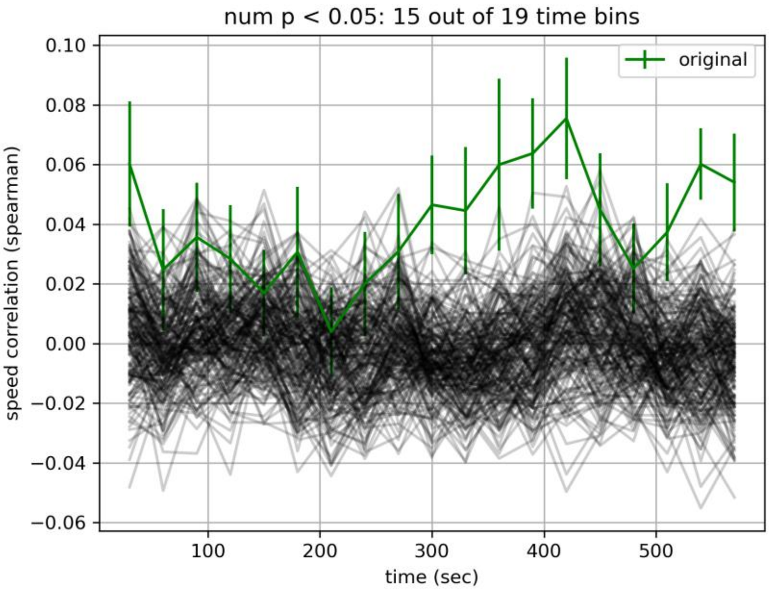

Figure S14. Moving window speed correlation between the children in our data (green) vs. shuffled distribution (black lines) of 200*23 random time series combinations of 3 children from three different videos. Fifteen out of 19 time-bins show an above chance speed correlation.

Importance of machine learning predictors

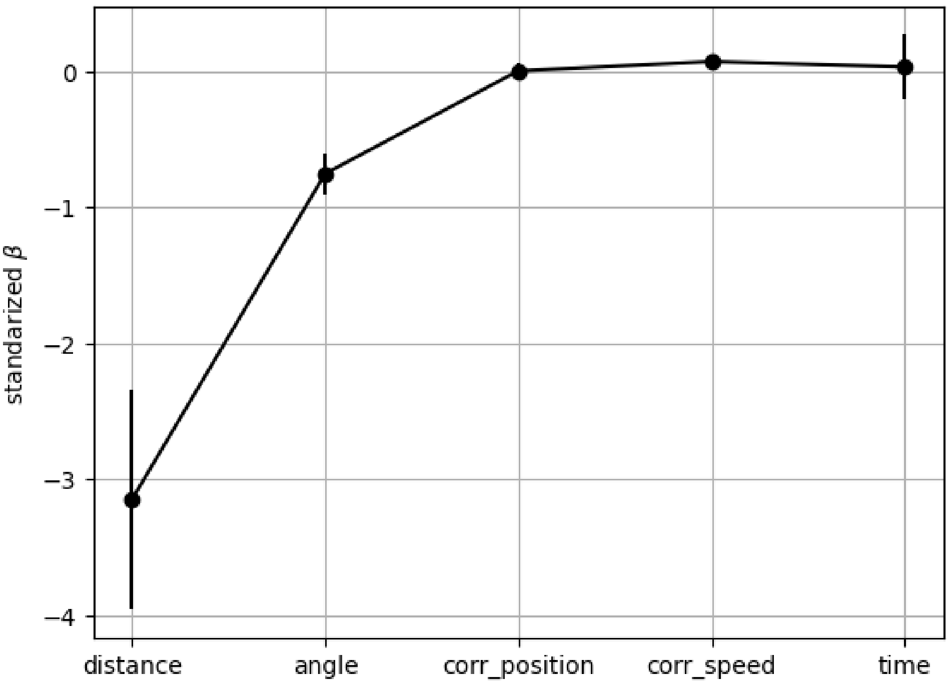

Figure S15. Predictor importance. Plotted importance of standardized coefficients in predicting social interactions with a logistic regression model. Fitted for each video separately, error bars depict 2 standard errors of the mean.

Machine learning with only “clear” social interactions

We replicated the machine learning analyses, but with only instances of social interactions in the sub-category “clear” as the outcome variable (i.e., children were within reaching distance of each other, oriented towards each other, and the coder was confident that a social interaction took place). This analysis resulted in higher overall accuracies, and stronger predictive power of distance and social orientation alone than in the machine learning model including all sub-categories of social interactions (see Table S5 for details).

Table S5. Machine learning model predicting “clear” social interactions.

	Training Accuracy %	Cross-Validation Accuracy %
Distance	77.6 (0.5)	71.5 (10.6)
Social Orientation	64.5 (0.3)	61.0 (5.6)
Positional correlation	50.9 (0.2)	50.2 (1.9)
Speed correlation	52.2 (0.1)	54.6 (2.0)
Time	51.6 (0.6)	49.2 (6.2)
Overall	81.8 (0.5)	77.3 (9.6)
Overall (Random Forest)	81.9 (0.4)	74.6 (10.2)

Note. Accuracy values represent means, with standard deviations across folds in parentheses.

Dyadic and group-level variations in social interactions and automated measures

Table S6. Model results for the outcome variable social interaction duration

Fixed Effects					
	Est/Beta	SE	95% CI	*t*	*p*
Intercept	18.988	7.066	[5.457, 33.415]	2.687	0.014
Group composition (NF)	4.069	8.770	[-15.485, 21.286]	0.464	0.649
Dyad type (non-friend)	-15.400	6.412	[-28.161, -4.201]	-2.402	0.024
Time bin	-0.053	0.148	[-0.312, 0.271]	-0.356	0.722
**Random Effects**					
				Variance	SD
Dyad ID (Intercept)				375.3	19.4
Observation (Intercept)				426.2	20.7
Residual				214.6	14.7
**Model Fit**					
				Marginal	Conditional
R^2				0.033	0.796

Key: LMM fitted using REML and statistical significance was assessed using Satterthwaite's method (package lmerTest), with adjusted degrees of freedom reported. Confidence intervals for fixed effects were calculated using the bootstrap method of package lme4.

Model equation: dyadSocIntDur_s ~ videoCondition + dyadType + win_centre_min_scaled + (1 | dyadID) + (1 | video)

Table S7. Model results for the outcome variable distance

Fixed Effects					
	Est/Beta	SE	95% CI	*t*	*p*
Intercept	990.903	80.831	[831.560, 1162.056]	12.259	<0.001
Group composition (NF)	-174.712	112.723	[-398.255, 27.091]	-1.550	0.1367
Dyad type (non-friend)	202.754	56.893	[90.244, 333.010]	3.564	0.001
Time bin	-0.197	3.493	[-7.069, 7.101]	-0.057	0.955
**Random Effects**					
				Variance	SD
Dyad ID (Intercept)				19688	140.3
Observation (Intercept)				67187	259.2
Residual				119961	346.4
**Model Fit**					
				Marginal	Conditional
R^2				0.023	0.433

Key: LMM fitted using REML and statistical significance was assessed using Satterthwaite's method (package lmerTest), with adjusted degrees of freedom reported. Confidence intervals for fixed effects were calculated using the bootstrap method of package lme4.
Model equation: dyadDist ~ videoCondition + dyadType + win_centre_min_scaled + (1 | dyadID) + (1 | video)

Table S8. Model results for the outcome variable social orientation.

Fixed Effects					
	Est/Beta	SE	95% CI	*t*	*p*
Intercept	0.338	0.011	[0.316, 0.357]	31.331	<0.001
Group composition (NF)	-0.011	0.014	[-0.037, 0.021]	-0.779	0.446
Dyad type (non-friend)	0.023	0.012	[0.000, 0.049]	1.942	0.058
Time bin	0.003	0.001	[0.001, 0.004]	3.434	0.001
**Random Effects**					
				Variance	SD
Dyad ID (Intercept)				0.001	0.028
Observation (Intercept)				0.001	0.030
Residual				0.006	0.075
**Model Fit**					
				Marginal	Conditional
R^2				0.015	0.247

Key: LMM fitted using REML and statistical significance was assessed using Satterthwaite's method (package lmerTest), with adjusted degrees of freedom reported. Confidence intervals for fixed effects were calculated using the bootstrap method of package lme4.

Model equation: dyadOrientation ~ videoCondition + dyadType + win_centre_min_scaled + (1 | dyadID) + (1 | video)

Table S9. Model results for the outcome variable position correlation.

Fixed Effects					
	Est/Beta	SE	95% CI	*t*	*p*
Intercept	0.113	0.031	[0.051, 0.177]	3.655	0.001
Group composition (NF)	0.060	0.043	[-0.023, 0.144]	1.384	0.178
Dyad type (non-friend)	-0.134	0.049	[-0.243, -0.037]	-2.751	0.008
Time bin	0.003	0.004	[0.005, 0.010]	0.736	0.462
Random Effects					
				Variance	SD
Dyad ID (Intercept)				0.010	0.010
Observation (Intercept)				0.004	0.064
Residual				0.153	0.391
Model Fit					
				Marginal	Conditional
R^2				0.013	0.096

Key: LMM fitted using REML and statistical significance was assessed using Satterthwaite's method (package lmerTest), with adjusted degrees of freedom reported. Confidence intervals for fixed effects were calculated using the bootstrap method of package lme4.
Model equation: atanh(positionCorr) ~ videoCondition + dyadType + win_centre_min_scaled + (1 | dyadID) + (1 | video)

Table S10. Model results for the outcome variable speed correlation.

	Est/Beta	SE	95% CI	*t*	*p*
**Fixed Effects**					
Intercept	0.033	0.001	[0.012, 0.055]	3.065	0.005
Group composition (NF)	0.060	0.015	[0.030, 0.090]	3.871	<0.001
Dyad type (non-friend)	-0.056	0.016	[-0.089, -0.029]	-3.527	<0.001
Time bin	0.003	0.001	[0.000, 0.006]	2.174	0.030
**Random Effects**					
				Variance	SD
Dyad ID (Intercept)				0.001	0.030
Observation (Intercept)				0.001	0.024
Residual				0.018	0.135
**Model Fit**					
				Marginal	Conditional
R^2				0.100	0.026

Key: LMM fitted using REML and statistical significance was assessed using Satterthwaite's method (package lmerTest), with adjusted degrees of freedom reported. Confidence intervals for fixed effects were calculated using the bootstrap method of package lme4.
Model equation: atanh(speedCorr) ~ videoCondition + dyadType + win_centre_min_scaled + (1 | dyadID) + (1 | video)

Effects of group composition on friend dyad dynamics

Table S11. Model results for the outcome variable social interaction duration.

Fixed Effects					
	Est/Beta	SE	95% CI	*t*	*p*
Intercept	18.788	7.414	[4.387, 32.991]	2.534	0.022
Group composition (NF)	4.703	8.645	[-12.642, 20.255]	0.544	0.595
Time bin	-0.301	0.230	[-0.711, 0.172]	-1.310	0.191
**Random Effects**					
				Variance	SD
Observation (Intercept)				399.9	20.0
Dyad ID (Intercept)				194.0	13.9
Residual				217.7	14.8
**Model Fit**					
				Marginal	Conditional
R^2				0.007	0.734

Key: LMM fitted using REML and statistical significance was assessed using Satterthwaite's method (package lmerTest), with adjusted degrees of freedom reported. Confidence intervals for fixed effects were calculated using the bootstrap method of package lme4.
Model equation: dyadSocIntDur_s ~ videoCondition + win_centre_min_scaled + (1 | dyadID) + (1 | video)

Table S12. Model results for the outcome variable distance.

Fixed Effects					
	Est/Beta	SE	95% CI	*t*	*p*
Intercept	982.701	128.937	[732.900, 1233.534]	7.622	<0.001
Group composition (NF)	-174.709	154.522	[-489.657, 120.901]	-1.131	0.276
Time bin	1.754	5.003	[-8.317, 12.692]	0.351	0.726
**Random Effects**					
				Variance	SD
Observation (Intercept)				126134	355.2
Dyad ID (Intercept)				51804	227.6
Residual				103441	321.6
**Model Fit**					
				Marginal	Conditional
R^2				0.025	0.642

Key: LMM fitted using REML and statistical significance was assessed using Satterthwaite's method (package lmerTest), with adjusted degrees of freedom reported. Confidence intervals for fixed effects were calculated using the bootstrap method of package lme4.

Model equation: dyadDist ~ videoCondition + win_centre_min_scaled + (1 | dyadID) + (1 | video)

Table S13. Model results for the outcome variable social orientation.

Fixed Effects					
	Est/Beta	SE	95% CI	*t*	*p*
Intercept	0.341	0.014	[0.314, 0.370]	24.405	<0.001
Group composition (NF)	-0.015	0.015	[-0.046, 0.017]	-1.006	0.343
Time bin	0.003	0.001	[0.001, 0.006]	2.935	0.003
**Random Effects**					
				Variance	SD
Observation (Intercept)				0.00096	0.03097
Dyad ID (Intercept)				0.00094	0.03074
Residual				0.00577	0.07596
**Model Fit**					
				Marginal	Conditional
R^2				0.018	0.262

Key: LMM fitted using REML and statistical significance was assessed using Satterthwaite's method (package lmerTest), with adjusted degrees of freedom reported. Confidence intervals for fixed effects were calculated using the bootstrap method of package lme4.

Model equation: dyadOrientation ~ videoCondition + win_centre_min_scaled + (1 | dyadID) + (1 | video)

Table S14. Model results for the outcome variable position correlation. Note that this model had a singular fit, indicated by the zero variance for random effect dyadID. For this reason, model fit with random effects cannot be calculated (conditional R^2 is NA).

Fixed Effects					
	Est/Beta	SE	95% CI	*t*	*p*
Intercept	0.104	0.047	[0.009, 0.196]	2.231	0.040
Group composition (NF)	0.067	0.068	[-0.073, 0.206]	0.992	0.334
Time bin	-0.007	0.006	[-0.019, 0.006]	-1.133	0.258
**Random Effects**					
				Variance	SD
Observation (Intercept)				0.018	0.134
Dyad ID (Intercept)				0.000	0.000
Residual				0.160	0.400
**Model Fit**					
				Marginal	Conditional
R^2				0.009	NA

Key: LMM fitted using REML and statistical significance was assessed using Satterthwaite's method (package lmerTest), with adjusted degrees of freedom reported. Confidence intervals for fixed effects were calculated using the bootstrap method of package lme4.
Model equation: atanh(positionCorr) ~ videoCondition + win_centre_min_scaled + (1 | dyadID) + (1 | video)

Table S15. Model results for the outcome variable speed correlation.

Fixed Effects					
	Est/Beta	SE	95% CI	*t*	*p*
Intercept	0.020	0.017	[-0.015, 0.053]	1.180	0.260
Group composition (NF)	0.066	0.016	[0.036, 0.098]	4.190	0.002
Time bin	0.001	0.002	[-0.003, 0.006]	0.553	0.581
**Random Effects**					
				Variance	SD
Observation (Intercept)				0.00049	0.02221
Dyad ID (Intercept)				0.00193	0.04399
Residual				0.01942	0.13937
**Model Fit**					
				Marginal	Conditional
R^2				0.046	0.152

Key: LMM fitted using REML and statistical significance was assessed using Satterthwaite's method (package lmerTest), with adjusted degrees of freedom reported. Confidence intervals for fixed effects were calculated using the bootstrap method of package lme4.

Model equation: atanh(speedCorr) ~ videoCondition + win_centre_min_scaled + (1 | dyadID) + (1 | video)
